# Chronotype and associations with dietary intake, meal timing, body composition, and metabolic biomarkers

**DOI:** 10.3389/fnut.2026.1862060

**Published:** 2026-07-07

**Authors:** Carlien van der Merwe, Marilize Richter-Cottle, Bernhard H. Breier, Jeroen Douwes, Mirjam Münch, Rozanne Kruger

**Affiliations:** 1 School of Sport, Exercise and Nutrition, Massey University, Auckland, New Zealand; 2 Centre of Excellence for Nutrition, North-West University, Potchefstroom, South Africa; 3 Centre for Public Health Research, Massey University, Wellington, New Zealand; 4 Centre for Chronobiology, Psychiatric Hospital of the University of Basel; Research Cluster Molecular and Cognitive Neurosciences; Department for Biomedicine, University of Basel, Basel, Switzerland; 5 School of Allied Health, Sport and Social Work, Griffith University, Gold Coast, QLD, Australia

**Keywords:** body fat percentage, circadian, evening types, meal composition, midsleep, obesity

## Abstract

**Background and aims:**

In humans, meals are important non-photic zeitgebers for the circadian timing system, which is also influenced by chronotype. This study assessed whether chronotype is associated with dietary intake (energy; nutrients), meal timing, body composition markers, and metabolic biomarkers in healthy European and Pacific New Zealand (NZ) women.

**Methods:**

Whole-body fat percentage (BF%), android-to-gynoid (AG) fat percentage ratio were assessed in 287 healthy non-pregnant European and Pacific NZ women aged 18–45 years using dual-energy X-ray absorptiometry (DXA). Body mass index (BMI) was calculated, and chronotype was assessed utilizing the Munich Chronotype Questionnaire (MCTQ). A 5-day estimated food record was used to assess dietary intake. Fasting venous blood samples were collected to evaluate metabolic biomarkers.

**Results:**

The majority of participants (54%) were classified as intermediate chronotypes (IT), 34% as evening types (ET), and 12% as morning types (MT). The ET group had higher BMI, BF%, and AG fat percentage ratio than the morning and intermediate-chronotypes groups (MT-IT): 31.4 vs. 26.1 kg/m^2^, 36 vs. 34%, and 0.98 vs. 0.87, respectively. Daily energy intake was higher in ET than in MT-IT (*p* = 0.03). MT-IT had higher intakes of energy, protein, carbohydrate, and fat in the morning than ET (before 10:00); in contrast, ET had higher energy, protein, carbohydrate, and fat intakes in the evening after 20:00. The ET in high BF% and high AG ratio groups were more likely to have lower energy, protein, and carbohydrate intakes before 10:00 in the morning, as well as higher energy, carbohydrate, and fat intakes after 20:00. The ET group had overall a poorer lipid profile as well as glucose homeostasis than MT-IT (all *p* < 0.05).

**Conclusion:**

The ET women consumed less energy and less micronutrient-dense food than the MT-IT. The ET had lower dietary intake in the morning but higher intake in the evening, with an inverse pattern observed among MT-IT. Higher energy and macronutrient intake in the evening was significantly associated with a higher BF% and AG ratio in ET, suggesting a potentially greater susceptibility to obesity.

## 1 Introduction

Humans tend to structure the timing of their sleep and wake episodes within the 24-h day according to their subjective preferences. These individual preferences for sleep–wake timing relative to the light–dark cycle are referred to as chronotype (1). A person’s chronotype influences the timing of diurnal activity preferences and the modulation of physiological functions and behavior (2–4). The sleep and wake times in morning types (MT) are several hours earlier than in evening types (ET) (5). Chronotype influences not only sleep and wake times (6) but also food intake timing. The majority of the studies examining associations between chronotype and dietary intake have focused on absolute food intake (7–15), with some also looking at the distribution of energy (16–21) and macronutrient intakes (17, 20, 22, 23) throughout the day. Recent systematic reviews found that both energy intake and macronutrient intake, and their distribution between the morning and evening, varied between chronotypes. Further, late types usually consumed less healthy food, had unhealthy eating habits and patterns, and had a greater risk of higher body weight and body mass index (BMI) (24, 25). However, evidence on how energy and macronutrient distribution across the day relates to other body composition markers beyond BMI remains limited, especially in the New Zealand (NZ) population. Culnan et al. (26) found that the time between the last meal and dim light melatonin onset (DLMO) in the evening was not associated with higher BMI, except for those who had their last meal after 10.30 p.m.

Since the majority of the food-related metabolic processes, such as appetite, food intake, digestion, nutrient metabolism, and hormonal regulation, are under circadian control (27, 28), macronutrient metabolism and energy expenditure change across the 24-h light–dark cycle (29, 30), exhibiting a marked morning-evening difference (31–35). As a result, not only the timing of food intake but also variations in energy and macronutrient intake and their distribution throughout the day affect energy expenditure (36–38). Collectively, these may affect body composition and metabolic biomarker outcomes differently across chronotypes.

Few studies have assessed the effects of energy and macronutrient distribution on body composition (assessed using BMI) across chronotypes, with mixed results (20, 23). Body mass index is a useful measure of adiposity in population studies for addressing obesity-associated health risks (39). However, it is not a suitable measure for reliably determining muscle mass or fat mass (FM) and associated metabolic disease risk at the individual level, especially if BMI is not in the obese categories (39, 40). For example, in NZ, Pacific women typically have larger body size and higher fat-free mass (FFM) than NZ European (NZE) women (41). Therefore, total body fat percentage (BF%) (39, 42), android-to-gynoid (AG) fat percentage ratio, and visceral fat percentage are better indicators of metabolic health (43) and adiposity (19, 44–46) in these groups. Previous studies have demonstrated associations between a poor lipid profile (higher triglycerides and lower high-density-lipoprotein (HDL)-cholesterol concentrations) (20, 45) and glucose homeostasis (45) among those identified as ET, suggesting a connection between chronotype and metabolic health outcomes.

It is plausible that food should merely be consumed during “daytime” hours and limited during “nighttime” hours, since both energy and nutrients are optimally metabolized during the daytime (29–35, 47–50). However, it remains to be established whether a link exists among chronotype, diet, body composition, and biomarkers. The aim of this study was to assess whether chronotype is associated with dietary intake (energy and nutrients), meal timing across the day, various body composition markers, and metabolic biomarkers in healthy European and Pacific NZ women.

## 2 Materials and methods

### 2.1 Study design, participants, and procedure

This study used data from a large cross-sectional study (PRedictors linking Obesity and the gut MIcrobiomE [PROMISE] study) that recruited and collected data from 304 healthy women from Auckland, NZ (51). This was an exploratory secondary analysis identifying and investigating different chronotypes among participants who met the inclusion criteria of Pacific and NZE ethnicity, ages between 18 and 45, and either a BMI < 25 (normal) or ≥ 30 kg/m^2^ (obese) (51). The initial screened BMI was based on self-reported height and weight. Exclusion criteria were pregnancy/lactation; diagnosis of chronic illnesses (e.g., diabetes and cardiovascular disease); previous bariatric surgery; severe food allergies; using medication that could interfere with appetite or the immune system (e.g., appetite suppressants and corticosteroids); current smokers or with severe dietary restrictions or avoidances (e.g., vegan).

Written informed consent was obtained from all participants. Ethics approval was granted by the Southern Health and Disability Ethics Committee, NZ (16/STH/32), and the trial was registered at anzctr.org.au (ACTRN12618000432213). Participants visited the Massey University Human Nutrition Research Unit in Auckland, NZ, between July 2016 and September 2017 twice for data collection, up to 14 days apart. The first data collection included fasted blood sampling (metabolic biomarkers), body composition measurements (height, weight, and BF analysis using bioelectrical impedance analysis (BIA), completing the Munich Chronotype Questionnaire (MCTQ), a retrospective 7-day sleep and work diary/questionnaire, and an interview to collect demographic information.

Following, at-home data collection included a 5-day estimated food record (5DFR) and an accelerometer protocol to objectively measure physical activity and sleep (results of these analyses are reported elsewhere (52)). The second data collection returned accelerometers, diaries, and the 5DFR (with recipes and label information), which were reviewed by a dietitian for completeness, followed by an individual interview to clarify food intake data from the 5DFR. Further body composition measurements (weight, waist circumference [WC], hip circumference [HC], dual-energy X-ray absorptiometry [DXA] measurements) were performed. The study design and other measurements and data collected (e.g., stool samples) are reported in Kindleysides et al. (51). Procedures for this secondary analysis are described below.

### 2.2 Demographic information and anthropometric measurements

Demographic (e.g., age, address, and work patterns) and health (e.g., disease conditions and medication use) information was collected during a standardized individual interview with a researcher during the first data collection. All anthropometric measurements were conducted using the International Society for the Advancement of Kinanthropometry (ISAK) protocols by ISAK-trained researchers (53). Height and weight were used to calculate the Quetelet index (weight/height^2^) to determine BMI. Waist and hip circumferences were measured with a Lufkin W600PM flexible steel tape with the participant in a relaxed standing position with their arms folded across their chest, to determine waist-to-hip ratio (WHR) and waist-to-height ratio (WHtR), respectively. Whole body DXA scans (Hologic QDR Discovery A, Hologic, Inc., with APEX V. 3.2 software) were performed to assess lean body mass (kg), whole body total fat mass (kg), android-, gynoid-, and visceral-fat percentage, and android-to-gynoid (AG) fat percentage ratio (39). Both FM and FFM were measured by DXA and BIA and used to determine the FM: FFM ratio.

### 2.3 Chronotype, sleep, and work schedules

A retrospective sleep-and-work questionnaire was used to collect information on sleep and work schedules over the last 7 days, including the number of hours worked per day and whether day or night shifts were worked. Chronotype was subjectively assessed using the English version of the MCTQ, developed by Roenneberg et al. (1), based on self-reported sleep timing (bed and wake times and estimated time to fall asleep) on workdays and work-free days within the last 4 weeks (54). The questionnaire was completed online (SurveyMonkey, Inc., United States) during the first data collection. Midsleep (the midpoint time between self-reported sleep onset and wake time of the last 4 weeks) was calculated from habitual sleep times. It was determined separately for workdays, that is, midsleep on workdays (MSW) and midsleep on work-free days (MSF), then weighted for midsleep on free days and corrected for sleep duration on free days (MSFsc) (1). The MSFsc was used as a proxy for chronotype, where scores >5 (i.e., midsleep time occurs after 05:00) were considered ET and scores <3 (i.e., midsleep time occurs before 03:00) were considered MT. Habitual MSFsc times >2.99 and ≤5 were considered intermediate (IT) chronotypes.

### 2.4 Dietary intake

Estimated, non-consecutive, 5-day food records (2 weekend-days and 3 weekdays) were used to analyze dietary intake for energy, macronutrients, micronutrients, and the distribution of food intake throughout the day (clock times). Participants received instructions for estimating and recording their food intake and portion sizes. Study dietitians conducted an in-depth food-record interview with all participants to assess the accuracy of portion sizes, cooking methods, and brands of reported food products. Various visual aids were used to confirm the portion sizes and brands of foods consumed, including a visual portion book, household measures (e.g., metric cups and spoons), and web-based tools (51). Data were entered manually into the computer software, FoodWorks 10 (Xyris Software (Australia) Pty Ltd., Queensland, Australia), and the NZ food composition database (NZ FOODFiles 2016) was used to analyze the energy, macronutrient, and micronutrient content of the 5DFR (51). Because of the vast array of food items consumed, if a match for any item could not be found in the NZ databases, food composition data were obtained from AusFoods 2017 (AUSNUT 2011–13 food composition files), developed by Food Standards Australia New Zealand. Energy intake cut-offs of <2,100 kJ/day and >27,000 kJ/day identified under and over-reporters, with upper limits extended as in prior studies on ethnic minority groups (55). Overall, 17 women (16 Pacific; 1 NZE) were excluded from the final sample due to over-reported energy intakes >27,000 kJ/day.

### 2.5 Blood

Fasting venous blood samples were collected during the first data collection after an overnight fast of ≥10 h. Blood was drawn into 4 vacutainers (each with a maximum volume of 30 mL) to obtain plasma and serum samples. Whole blood samples were drawn into ethylenediaminetetraacetic acid (EDTA) 10 mL vacutainers (Becton Dickinson) and stored immediately at −80 °C. Plasma samples were collected in a 2 mL Becton Dickinson vacutainer P800 EDTA, aprotinin, and dipeptidyl peptidase IV. For serum samples, the vacutainer (10 mL, Becton Dickinson) was kept at room temperature (18 °C) for 30–60 min to allow clotting before centrifugation. All 4 vacutainers were immediately placed on wet ice after collection, and standard procedures were followed before centrifugation at 1,500 *g* for 15 min at 4 °C within 1 h of collection. Plasma and serum samples were frozen at −80 °C until analysis at the Liggins Institute, Auckland, New Zealand. Metabolic biomarkers related to lipid profiles (triglycerides, HDL, low-density-lipoprotein (LDL), and total cholesterol), glucose (plasma glucose, insulin, and glycated hemoglobin), and hormonal control (ghrelin, leptin, C-reactive protein, and Peptide YY), were analyzed. For homeostasis model assessment (HOMA-IR), the index for insulin resistance was calculated by using the formula: [fasting blood glucose (mmol/L) × fasting plasma insulin (μU/mL)/22.5] (51).

### 2.6 Data analysis

**Figure 1:**
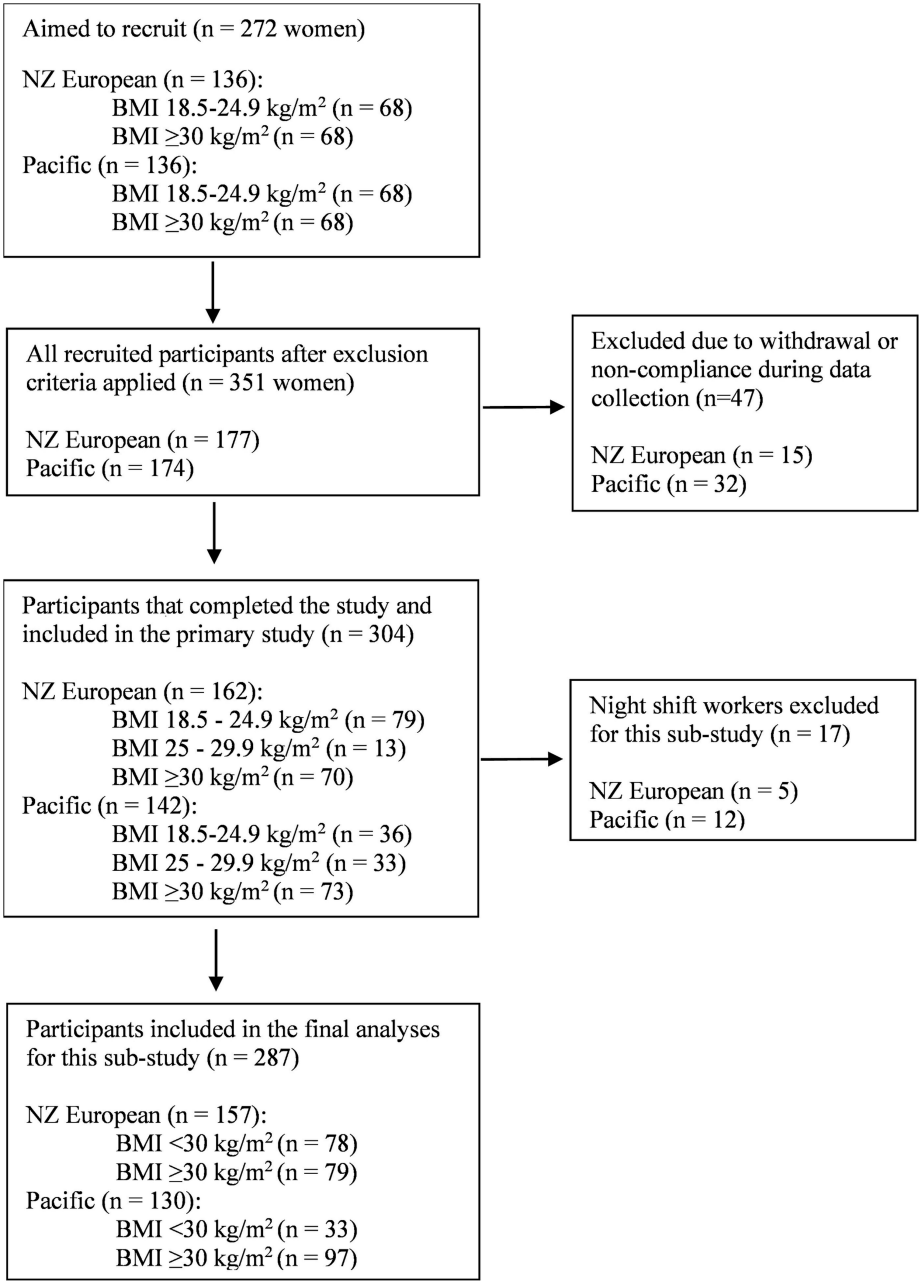
Flowchart of recruited participants included in this substudy. NZ, New Zealand European; BMI, body mass index.

Analyses were conducted using IBM Statistical Package for the Social Sciences (SPSS) Statistics for Windows version 28 (IBM Corp, Armonk, NY, United States), and a *p*-value of <0.05 was considered statistically significant. Night-shift workers (*n* = 17) were excluded from the analysis because their altered sleep–wake and fasting-feeding cycles are not necessarily representative of their chronotype. The final analysis comprised 287 women, including 157 NZE and 130 Pacific women (Figure 1).

To assess the association between chronotype, timing of dietary intake, body composition, and biomarkers, the study participants were grouped as ET (MSFsc > 5), IT (MSFsc = 3–5), and MT (MSFsc < 3). Since the study was not *a priori* designed to include equal chronotype groups, and because this age group is expected to consist mainly of intermediate or late chronotypes with only a few morning types (56), the MT and IT groups were merged into a single MT–IT group due to the small sample size of the MT group (14.6%). The rationale for merging IT with MT, rather than ET, was based on the relative importance of preserving ET for statistical assessments, given ET’s greater metabolic involvement in the literature due to its greater susceptibility to circadian misalignment (57). Since some participants’ estimated self-reported height and weight obtained during recruitment did not match the target BMI categories following actual measurements during the data collection period (i.e., including > 25 and < 30 kg/m^2^), we instead stratified participants according to collected body composition data. These included two BMI groupings: normal < 25 kg/m^2^ and high ≥ 25 kg/m^2^ BMI, thus collapsing women with overweight and obese BMI into one group and women with normal BMI into another group; two BF% groupings: normal BF < 35% and high BF ≥ 35% as defined by the Obesity Medicine Association (58); and 2 android-to-gynoid (AG) fat percentage ratio groupings: normal < 0.8 or high ≥ 0.8 AG ratio (59). We were unable to stratify for ethnicity due to low numbers in the various chronotype groups and therefore adjusted for ethnicity during analysis. The NZ deprivation Index 2013 (NZDep2013), based on geographical residential area, was used as a measure of socioeconomic status. Scores range from 1 to 10, where 1 represents “least deprived” and 10 “most deprived” areas (60).

The distribution of the variables used was assessed using the Kolmogorov–Smirnov and Shapiro–Wilk tests, box plots, and histograms. Normally distributed data were presented as means and standard deviations; non-normally distributed data as medians and 25th and 75th percentiles; and categorical data as frequency summary statistics. Log-transformed data values were back-transformed to the original scale and reported as geometric mean and geometric standard deviation. Welch’s *t*-tests were used for normally distributed data, and Mann–Whitney U tests for non-normal data when comparing two groups. When comparing more than two groups, analysis of variance (ANOVA) tests (with Tukey HSD as *post hoc* tests) were used for normally distributed data, and Kruskal–Wallis tests (with multiple comparison rank tests, adjusted by Bonferroni correction) for non-normally distributed data.

To create food intake time windows, we used a data-driven approach. First, the energy distribution over 24 h was binned into hourly food intake for the two chronotype groups (MT-IT and ET). Statistical significance between chronotypes at each hour was determined using multiple comparison rank tests, adjusted for a Bonferroni correction (see Supplementary Figure S1). Based on this distribution, and usual eating patterns of the NZ population, as well as previous studies, we defined four dietary windows: a late night/early morning window (7-h range): 03:00–09:59 h, a late morning/early afternoon window (5-h range): 10:00–14:59, a late afternoon/early evening window (5-h range): 15:00–19:59 h, and a late evening/early night window (7-h range): 20:00–02:59 h. This approach aligns with food intake categories created in other studies, although all used slightly different “windows” (17, 21, 22). Mean intakes per participant across the five measurement days were determined for each time window. A two-way repeated measures analysis of covariance (ANCOVA) was performed to compare the effect of chronotype on the distribution of mean energy and macronutrient intakes across dietary time windows (using Greenhouse–Geisser estimates of sphericity) with Bonferroni adjustments applied. The analysis was adjusted for ethnicity (NZE, Pacific), age, and deprivation index. Pearson’s correlation analysis was conducted to assess associations between MCTQ (using continuous individual midsleep times from all participants), energy intake at different times, biomarkers, and body composition markers.

The differences for nutrient intake between chronotypes in the late night/early morning window, (i.e., before 10:00 h) and the late evening/early night window (i.e., after 20:00 h) were assessed by multiple linear regression models within normal (<35%) and high BF% (≥35%) groups, and then within normal (<0.8) and high (≥ 0.8) AG ratio groups to denote central adiposity. Each nutrient’s average intake per time window was used as the dependent variable and chronotype (with ET as reference category coded as 1 and MT-IT as non-reference category coded as 0) as the independent variable. Each model was adjusted for age, deprivation index, and ethnicity.

## 3 Results

### 3.1 Study participants and demographics

The descriptive statistics are shown separately for each of the three chronotype groups and for the combined MT-IT group in Table 1. The majority of ET were of Pacific ethnicity (*n* = 83, *p* < 0.001), whilst IT and MT were mostly NZE (*n* = 115 and *n* = 28, respectively; *p* < 0.01). The ET were younger than MT-IT, lived in more socioeconomically deprived areas, and had higher BMI, WC, HC, and android and visceral fat percentages (all *p* < 0.05; Table 1). As per the definition, the ET vs. MT had later wake times (09:48 vs. 06:48) and sleep onset (00:48 vs. 22:18) times, as well as greater social jetlag (defined as differences in habitual bed and wake times between workdays and free days (13)) (1.75 h vs. 0.77 h). The self-reported sleep duration was similar between ET and MT-IT (8.8 vs. 8.9 h, *p* = 0.63).

**Table 1:** Demographics, sleep–wake, and body composition characteristics.

Variable	MT (*n* = 35)	IT (*n* = 155)	ET (*n* = 97)	*p*-value ^**†**^	MT IT^**†**^ (*n* = 190)	*p-*value *^ƶ^*
Demographics						
Age (years)	35 (29, 38)	29 (24, 37) ^a^	23 (21, 26) ^a, b^	**0.03**	30.0 (28.9, 31.0)	**<0.01**
NZ European *n* (%)	28 (17.8)	115 (73.2)	14 (8.9)	**<0.01**	143 (49.8)	**<0.01**
Pacific *n* (%)	7 (5.4)	40 (30.8)	83 (63.8)		47 (16.4)	
Deprivation score	5 (2, 6)	5 (3, 7)	8 (6, 10) ^a, b^	**<0.01**	5 (3, 7)	**<0.01**
Body composition						
BMI (kg/m^2^)	24.2 (21.7, 31.4)	27.3 (22.7, 33.1)	31.4 (24.6, 36.8) ^a, b^	**<0.01**	26.1 (22.7, 32.9)	**<0.01**
BMI categories, *n* (%)						
<25 kg/m^2^	19 (54)	66 (43)	26 (27)	**0.01**	85 (45)	**<0.01**
≥25 kg/m^2^	16 (46)	89 (57)	71 (73)		105 (55)	
BF%	32 ± 9	34 ± 7	36 ± 7	**0.02** ^a Ɏ^	34 ± 7	**0.02** ^ɍ^
BF% categories, *n* (%)						
<35%	23 (66)	69 (45)	45 (46)	0.07	92 (48)	0.75
≥35%	12 (34)	86 (55)	52 (54)		98 (52)	
WC (cm)	78.2 (72.1, 96.9)	81.5 (73.8, 96.1)	88.8 (78.0, 102) ^a, b^	**0.01**	80.2 (73.2, 96.1)	**<0.01**
HC (cm)	102 (95.2, 117)	108 (99.7, 119)	114 (104, 124) ^a, b^	**<0.01**	107 (99.1, 119)	**<0.01**
Android fat (%)	29.5 (19.9, 39.4)	34.3 (25.5, 39.8)	37.0 (29.2, 42.7) ^a, b^	**0.01**	33.5 (24.3, 39.6)	**<0.01**
Gynoid fat (%)	35.7 ± 6.5	37.9 ± 5.7	38.8 ± 5.2 ^a^	**0.02** ^Ɏ^	37.5 ± 5.9	0.05^ɍ^
Visceral fat (%)	27.1 (17.1, 37.3)	32.2 (22.2, 38.3)	34.8 (27.6, 41.2) ^a, b^	**<0.01**	31.4 (21.5, 38.1)	**<0.01**
AG ratio	0.80 (0.69, 0.96)	0.87 (0.77, 0.98)	0.98 (0.81, 1.06) ^a, b^	**<0.01**	0.87 (0.74, 0.98)	**<0.01**
AG ratio groups *n* (%)						
<0.8	18 (51.4)	54 (34.8)	23 (23.7)	**<0.01**	72 (37.9)	**0.02**
≥ 0.8	17 (48.6)	101 (65.2)	74 (76.3)		118 (62.1)	
Sleep characteristics						
Average wake time (hh:mm)	06:48 (06:24, 07:12)	08:00 (07:30, 08:30) ^a^	09:48 (09:00, 10:54) ^a, b^	**<0.01**	07:48 (07:18, 08:30)	**<0.01**
Wake time free days (hh:mm)	07:30 (07:00, 08:00)	08:30 (08:00, 09:00) ^a^	10:30 (10:00, 11:30) ^a, b^	**<0.01**	08:00 (08:00, 09:00)	**<0.01**
Wake time workdays (hh:mm)	06:00 (05:45, 06:30)	07:30 (07:00, 08:00) ^a^	09:23 (08:00, 10:30) ^a, b^	**<0.01**	07:15 (06:30, 08:00)	**<0.01**
Average sleep onset (hh:mm)	22:18 (21:54, 22:30)	23:00 (22:42, 23:42) ^a^	00:48 (00:06, 01:36) ^a, b^	**<0.01**	22:54 (22:30, 23:30)	**<0.01**
Sleep onset Free days (hh:mm)	22:15 (22:00, 22:40)	23:30 (23:04, 00:04) ^a^	01:52 (01:10, 02:30) ^a, b^	**<0.01**	23:18 (22:43, 00:00)	**<0.01**
Sleep onset workdays (hh:mm)	22:04 (21:25, 22:34)	22:37 (22:07, 23:04) ^a^	23:42 (22:54, 00:42) ^a, b^	**<0.01**	22:30 (22:00, 23:00)	**<0.01**
Social jetlag	0.77 (0.29, 1.21)	0.92 (0.50, 1.25)	1.75 (1.00, 2.47) ^a, b^	**<0.01**	0.88 (0.50, 1.25)	**<0.01**
Average sleep duration (hrs)	8.5 (8.1, 9.3)	8.9 (8.4, 9.5)	8.8 (8.1, 9.9)	0.28	8.9 (8.3, 9.5)	0.63
Sleep duration free days (hh:mm)	9.07 ± 1.02	8.95 ± 1.07	8.71 ± 2.03	0.33	8.97 ± 1.06	0.24
Sleep duration workdays (hh:mm)	8.16 ± 1.06	8.82 ± 1.13	9.13 ± 2.22^a^	**<0.01**	8.69 ± 1.15	0.07

Values are median (25th and 75th percentiles), means ± standard deviation (SD), Missing deprivation index (*n* = 3); ^Ɏ^One-way ANOVA for comparing three groups of continuous, normally distributed data with Tukey HSD as post hoc test. †Kruskal–Wallis with multiple comparison rank tests were used to compare three groups (MT, IT, and ET) of continuous, non-normally distributed data ^a^compared against MT, ^b^compared against IT, 0.05/3 = 0.0167, adjusted by the Bonferroni correction for multiple comparisons: 0.05/3 = 0.0167; ^ƶ^Mann–Whitney Test for comparing two groups (ET, MT-IT) continuous non-normally distributed data; Welch’s independent t-test for comparing MT-IT with ET groups for continuous, normally distributed data. Chi-square tests were used to compare categorical data; significant *p* is shown in bold. MT-IT, morning and intermediate types combined group; MT, morning types; IT, intermediate types; ET, evening types; NZE, New Zealand European; BMI, body mass index, BF%, body fat percentage, WC, waist circumference, HC, hip circumference, AG, android-to gynoid fat percentage ratio.

### 3.2 Metabolic biomarkers and total dietary intake differences between chronotypes

Overall, ET had significantly higher concentrations of triglycerides, leptin, insulin, and hemoglobin A1C (HbA1C) than MT-IT. In contrast, total cholesterol, HDL and LDL-cholesterol, and ghrelin concentrations were higher in MT-IT than in ET (*p* < 0.05; Table 2).

**Table 2:** Metabolic biomarkers and dietary intake differences between chronotype groups.

Variable	MT (*n* = 35)	IT (*n* = 155)	ET (*n* = 97)	*p*-value^†^	MT-IT^†^ (*n* = 190)	*p-*value *^ƶ^*
Metabolic biomarkers						
Cholesterol (mmol/L)	5.07 ± 1.26	4.93 ± 1.20	4.56 ± 1.16 ^a, b^	**<0.01** ^Ɏ^	5.0 ± 1.0	**<0.01** ^ɍ^
LDL-cholesterol (mmol/L)	3.0 (2.4, 3.4)	3.1 (2.5, 3.6)	2.8 (2.4, 3.3)	0.06	3.0 (2.5, 3.6)	**0.02**
HDL-cholesterol (mmol/L)	1.7 (1.4, 2.0)	1.6 (1.3, 1.9)	1.4 (1.2, 1.7) ^a, b^	**<0.01**	1.6 (1.3, 1.9)	**<0.001**
TC: HDL	2.98 ± 1.35	3.11 ± 1.30	3.16 ± 1.30	0.534 ^Ɏ^	3.20 ± 0.95	0.557 ^ɍ^
LDL: HDL	1.80 ± 1.54	1.89 ± 1.48	1.91 ± 1.46	0.689 ^Ɏ^	2.03 ± 0.86	0.877 ^ɍ^
Triglycerides (mmol/L)	0.8 (0.7, 1.2)	0.9 (0.6, 1.1)	1.0 (0.8, 1.2) ^b^	**0.01**	0.8 (0.7, 1.1)	**<0.01**
HbA1c (mmol/L)	31.8 ± 2.7	31.6 ± 2.9	33.0 ± 3.1 ^b^	**0.01** ^Ɏ^	31.5 ± 1.09	**<0.01** ^ɍ^
Insulin (uU/ml)	8.4 (6.0, 10.1)	10.1 (6.7, 14.9)	15.7 (10.2, 26.3) ^a, b^	**<0.01**	9.5 (6.7, 14.7)	**<0.01**
Glucose (mmol/L)	5.2 (5.1, 5.3)	5.3 (5.3, 5.4)	5.4 (5.3, 5.5)	0.15	5.3 (5.0, 5.6)	0.16
Ghrelin (pg/mL)	74.8 (45.4, 120.8)	46.3 (22.1, 74.2) ^a^	28.1 (17.3, 58.7) ^a, b^	**<0.01**	48.9 (24.5, 83.6)	**<0.01**
Peptide YY (pg/mL)	120 (97, 148)	136 (114, 168)	135 (104, 168)	0.20	133 (111, 167)	0.78
Leptin (pg/mL)	7,210 (2,716, 14,683)	11,291 (5,726, 20,459)	15,678 (7,683, 23,196) ^a^	**0.01**	10,304 (4,895, 19,708)	**0.01**
C-reactive protein (mg/L)	0.9 (0.3, 2.2)	1.2 (0.5, 2.8)	0.8 (0.3, 2.9) ^b^	0.06	1.1 (0.5, 2.7)	0.10
Dietary intake						
Energy (kJ)	8,553 ± 1,702	8,527 ± 2,056	8,745 ± 2,577	0.74^Ɏ^	8,532 ± 1,992	**0.03** *^ɍ^*
Caffeine (mg)	117 (45.8, 282)	107 (37.9, 232)	54.4 (21.5, 120) ^a, b^	**<0.01**	108 (38.6, 234)	**<0.01**
Alcohol (g)	0.32 (0.01, 11.0)	0.09 (0.00, 7.64)	0.03 (0.00, 0.32) ^a^	**0.01**	0.09 (0.00, 7.64)	**0.01**
Macronutrients^$^						
Total Protein (g)	85.6 ± 20.3	86.5 ± 21.1	82.4 ± 24.4	0.36 ^Ɏ^	86.3 ± 20.9	0.17*^ɍ^*
Protein (%Energy)	17.2 ± 3.33	17.5 ± 3.47	16.3 ± 3.08	0.08 ^Ɏ^	17.5 ± 3.44	0.07 *^ɍ^*
Total carbohydrate (g)	196.9 ± 50.5	191.7 ± 63.9	223.1 ± 76.5 ^b^	**<0.01** ^Ɏ^	192.7 ± 61.6	**<0.01** ^***ɍ***^
Carbohydrate (%energy)	39.3 ± 6.80	38.3 ± 8.69	43.3 ± 6.47	1.00 ^Ɏ^	38.5 ± 8.36	**<0.001** *^ɍ^*
Total sugar (g)^#^	90.6 (65.8, 98.6)	77.5 (59.4, 97.7)	81.5 (62.5, 116)	0.28	79.0 (60.2, 98.3)	0.26
Fiber (g)	25.6 (17.3, 33.9)	22.4 (18.8, 27.2)	17.4 (14.2, 21.1) ^a, b^	**<0.01**	22.5 (18.6, 28.1)	**<0.01**
Total fat (g)	89.8 ± 26.7	93.6 ± 30.6	89.7 ± 28.5	0.55 ^Ɏ^	92.9 ± 29.9	0.31*^ɍ^*
Fat (%energy)	39.5 ± 7.47	41.3 ± 7.39	39.0 ± 5.41	0.12 ^Ɏ^	41.0 ± 7.42	**0.02** ^***ɍ***^
MUFA (g)	31.3 (23.3, 42.1)	34.9 (25.9, 43.3)	32.7 (26.6, 40.1)	0.48	34.3 (25.6, 43.0)	0.54
PUFA (g)	13.6 (8.82, 19.1)	12.1 (9.52, 14.9)	11.3 (8.96, 13.5) ^a^	0.045	12.2 (9.39, 15.5)	**0.02**
SFA (g)	30.9 (24.5, 42.6)	34.5 (26.8, 44.6)	33.6 (28.0, 43.6)	0.65	34.0 (26.2, 44.0)	0.96
Cholesterol (mg)	269 ± 1.79	293 ± 1.75	262 ± 1.61	0.24	289 ± 1.76	0.12*^ɍ^*
Micronutrients^$^						
Vitamin A equivalents (μg)	793 (587, 1,003)	819 (587, 1,008)	511 (323, 783) ^a, b^	**<0.01**	806 (587, 1,007)	**<0.01**
Thiamine (mg)	1.3 (1.0, 1.6)	1.2 (0.9, 1.5)	1.2 (0.9, 1.6)	0.62	1.2 (1.0,1.6)	0.93
Riboflavin (mg)	1.7 (1.5, 2.0)	1.8 (1.4, 2.2)	1.6 (1.2, 2.1) ^b^	**0.04**	1.8 (1.4, 2.2)	**0.01**
Niacin equivalents (mg)	36.1 (30.0, 43.5)	35.1 (29.7, 39.9)	34.5 (27.2, 42.0)	0.72	35.2 (29.8, 40.2)	0.58
Vitamin B6 (mg)	2.0 (1.6, 2.6)	2.2 (1.7, 2.7)	2.0 (1.5, 2.8)	0.68	2.2 (1.6, 2.7)	0.45
Folate (μg)	330 (260, 416)	334 (256, 427)	256 (180, 351) ^a, b^	**<0.01**	332 (256, 420)	**<0.01**
Vitamin B12 (μg)	3.7 (2.7, 4.3)	3.5 (2.7, 4.7)	3.8 (2.8, 4.8)	0.62	3.5 (2.7, 4.7)	0.35
Vitamin C (mg)	63.7 ± 2.04	62.4 ± 1.94	52.9 ± 2.30	0.18 ^Ɏ^	62.7 ± 1.95	0.83^ɍ^
Vitamin E (mg)	10.3 ± 1.47	9.27 ± 1.51	7.94 ± 1.52 ^a, b^	**<0.01** ^Ɏ^	9.3 ± 1.51	**<0.01**
Sodium (mg)	2,526 ± 1.30	2,524 ± 1.42	2,721 ± 1.49	0.25 ^Ɏ^	2,524 ± 1.40	0.06^ɍ^
Potassium (mg)	3,170 ± 809	2,987 ± 762	2,615 ± 832 ^a, b^	**<0.01** ^Ɏ^	3,021 ± 772	**<0.01** *^ɍ^*
Magnesium (mg)	354 (271, 442)	310 (265, 383)	262 (217, 301) ^a, b^	**<0.01**	312 (265, 387)	**<0.01**
Calcium (mg)	810 (669, 1,050)	809 (628, 980)	607 (491, 861)	**<0.01**	809 (635, 980)	**<0.01**
Iron (mg)	12.8 ± 1.31	11.1 ± 1.40	10.9 1.40^a^	0.047 ^Ɏ^	12.0 ± 3.8	0.29 *^ɍ^*
Zinc (mg)	10.9 (9.0, 12.9)	10.1 (8.6, 11.8)	9.6 (8.3, 12.0)	0.27	10.2 (8.8, 11.8)	0.19
Selenium (μg)	56.3 ± 1.49	60.7 ± 1.54	53.7 ± 1.61	0.09 ^Ɏ^	59.9 ± 1.53	0.06^ɍ^
Iodine (μg)	110 (85.0, 133)	93.8 (73.7, 115)	82.7 (58.6, 114) ^a, b^	**<0.01**	95.4 (74.4, 126)	**<0.01**

Values are median (25th and75th percentiles), means ± SD, or geometric mean and geometric ± SD for the log-transformed data values, back transformed to the original scale; †Kruskal–Wallis with multiple comparison rank tests were used to compare between three groups (MT, IT, and ET) of continuous, non-normally distributed data: ^a^compared against MT, ^b^compared against IT, 0.05/3 = 0.0167, adjusted by the Bonferroni correction for multiple comparisons: 0.05/3 = 0.0167; ^ƶ^Mann–Whitney U test for comparing two groups (ET and MT-IT) continuous non-normally distributed data; Chi-square test for categorical data; ^Ɏ^One-way ANOVA for comparing three groups continuous, normally distributed data with Tukey HSD as post hoc test; ^ɍ^Independent i-test for comparing two groups continuous, normally distributed data; ^$^Macronutrients include protein, carbohydrate, fat and all its subcategories; Micronutrients include all the vitamins and minerals; ^#^Total sugars include all free and added sugars; Significant *p* is shown in bold. MT, morning types; IT, intermediate types; ET, evening types; MT-IT, morning and intermediate types combined group; HbA1c, hemoglobin A1C; LDL, low-density lipoprotein; HDL, high-density lipoprotein; SFA, saturated fatty acids; PUFA, poly-unsaturated fatty acids; MUFA, mono-unsaturated fatty acids.

There were no statistically significant differences in daily macronutrient intakes between the MT-IT and ET, except for slightly higher carbohydrates (30 g) in ET. The ET also had a higher daily mean energy intake than MT-IT (difference: 213 kJ; *p* = 0.03). The MT-IT had higher median intakes of alcohol, fiber, caffeine, polyunsaturated fatty acids (PUFA), vitamin A, riboflavin, folate, potassium, magnesium, calcium, and iodine, as well as a higher mean vitamin E intake (*p* < 0.05; Table 2).

### 3.3 Dietary intake across four time-windows (24 h)

**Figure 2:**
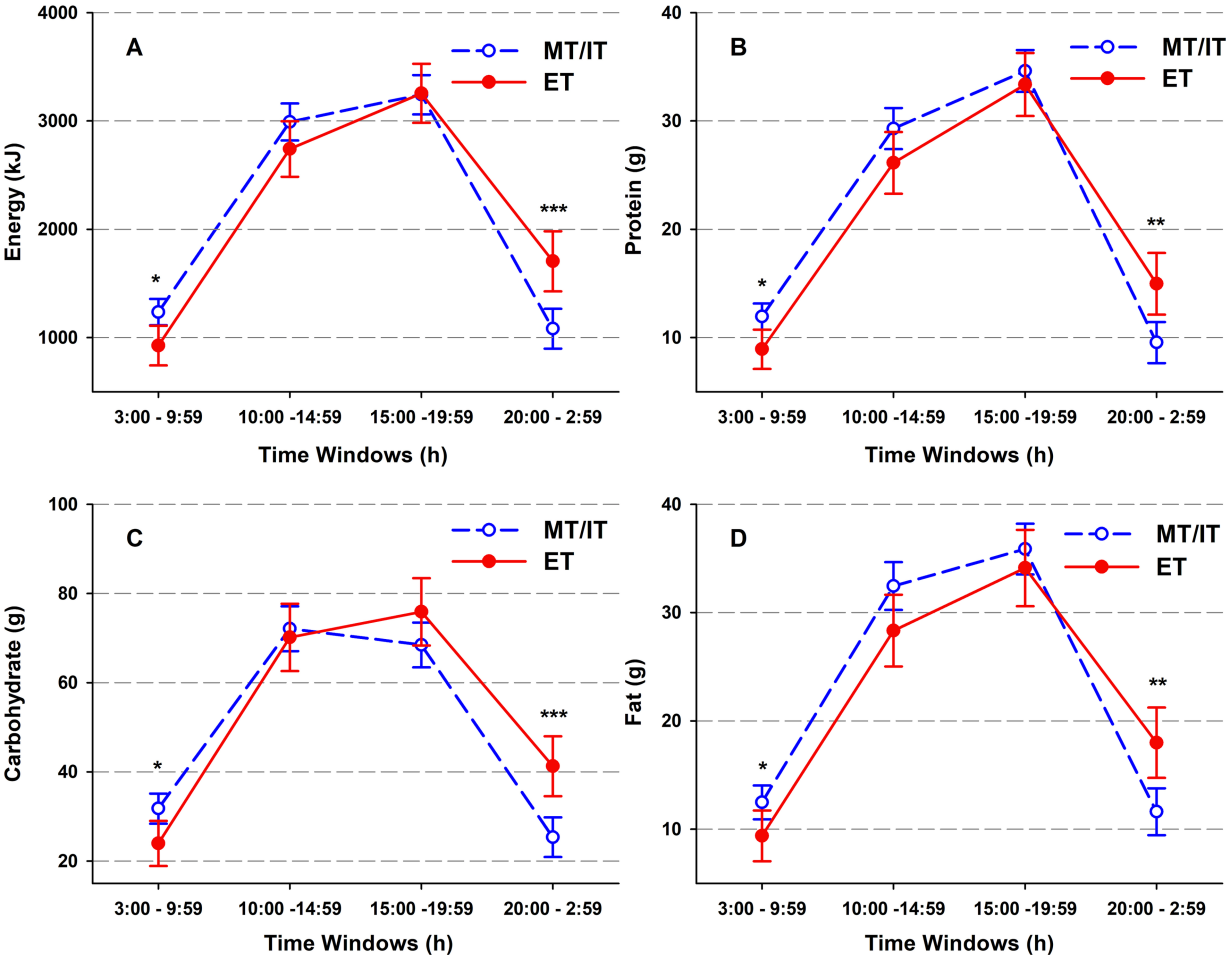
**(A–D)** the effect of chronotype and time window on the distribution of mean energy **(A)** and macronutrient **(B–D)** intakes. Time windows: Late night/early morning (03:00–9:59 h), late morning/early afternoon (10:00–14:59 h), late afternoon/early evening (15:00–19:59 h), late evening/early night (20:00–02:59 h). The results (estimated marginal means ± 2 SE) are shown for morning (MT; *n* = 35) and intermediate (IT; *n* = 155) chronotypes (combined into one group: MT/IT; blue symbols, dashed lines) and evening types (ET; *n* = 97; red symbols, solid lines) for: energy intake **(A)**, protein **(B)**, carbohydrate **(C)** and fat intake **(D)**. Two-way repeated measures ANCOVA was performed, adjusting for age, ethnicity, and deprivation index, with the within-subject factor as “time windows” and the between-subject factor as “chronotype.” Significant differences between chronotype groups:*p < 0.05; **p < 0.01; ***p < 0.001. Abbreviations: kilojoule (kJ), gram (g). Chronotypes: morning type (MT) and intermediate type (IT) were combined as a group (MT-IT), and evening type (ET).

Two-way repeated measures ANCOVA’s were performed to assess the effects of chronotype and the distribution of energy and macronutrient intakes across the 4 dietary intake time windows (see methods), while controlling for age, deprivation index, and ethnicity (Figures 2a–d).

Between the two chronotype groups, *post hoc* tests showed that MT-IT had statistically significantly higher energy (Figure 2A; *p* = 0.010), protein (Figure 2B; *p* = 0.012), carbohydrate (Figure 2c; *p* = 0.019), and fat (Figure 2d; *p* = 0.046) intake (g) than ET in the late night/early morning window. However, for ET, all these values were significantly higher than MT-IT in the late evening/early night window (*p* < 0.01; Figures 2a–d). For energy, protein, and carbohydrate intakes, conversely, there were no statistically significant differences between chronotype groups in the late morning/early afternoon as well as the late afternoon/early evening time windows (Figures 2a–d).

We also binned individual mealtimes across 24 h relative to habitual midsleep (MSFsc) in four equal 6-h time windows rather than to external clock times. We found no overall effect of chronotype on energy intake. However, there was a statistically significant interaction effect between chronotype and time (*p* = 0.007), indicating that 6 h past midsleep ET had a significantly higher intake (*p* = 0.006) than MT-IT and 24 h past midsleep vice versa (*p* < 0.001). The same result was found for protein and carbohydrates, but not for fat intake. MT-IT had significantly higher fat intake than ET at 18 and 24 h past midsleep (*p* = 0.041 and *p* < 0.001; see Supplementary Table S1 and Supplementary Figures S2A–D).

Similar analyses were performed for other nutrients across the 4 dietary intake time windows (Table 3). Overall, there was no significant main effect of chronotype on absolute intake of energy, protein, carbohydrate, or fat (kJ or g, respectively); however, there was a significant main effect of chronotype on carbohydrate intake expressed as %TE intake (Table 3). There was a significant main effect of time, as well as interaction between time and chronotype on energy intake [*F*(2.8,782) = 17.2, *p* < 0.001, partial ƞ^2^ = 0.06 and F(2.8,782) = 7.1, *p* < 0.001, partial ƞ^2^ = 0.03, respectively], protein intake [*F*(2.7,749) = 19.6, *p* < 0.001, partial ƞ^2^ = 0.07 and F(2.7,749) = 5.4, *p* = 0.002, partial ƞ^2^ = 0.02, respectively], carbohydrate intake [*F*(2.9,806) = 11.2, *p* < 0.001, partial ƞ^2^ = 0.04 and F(2.9,806) = 6.1, *p* < 0.001, partial ƞ^2^ = 0.02, respectively], and fat intake [*F*(2.9,798) = 12.3, *p* < 0.001, partial ƞ^2^ = 0.04 and F(2.9,798) = 5.9, *p* < 0.001, partial ƞ^2^ = 0.02, respectively].

**Table 3:** Distribution of dietary intake according to four time-windows (clock time) for different chronotype groups.

Dietary intake	CT (*n*)	Late N/early M (03:00–9:59 h)	Late M/early A (10:00–14:59 h)	Late A/early E(15:00–19:59 h)	Late E/early N (20:00–02:59 h)	*p*-values; main effects		
						Time	CT	Time*CT
Energy (kJ)	MT-IT (189)	1,236 [1,116, 1,355] SE 60.77^a^	2,991 [2,823, 3,158] SE 85.12^b^	3,242 [3,064, 3,420] SE 90.59^b^	1,081 [900, 1,263] SE 92.16^a^	<0.001	0.827	<0.001
	ET (95)	926 [746, 1.106] SE 91.37^a^	2,741 [2,489, 2.993] SE 127.98^b^	3.254 [2.986, 3.523] SE 136.21^c^	1.705 [1.432; 1.977] SE 138.57^d^			
*p*-value		**0.010**	0.138	0.944	**<0.001**			
Protein (g)	MT-IT (189)	11.94 [10.75, 13.13] SE 0.60^a^	29.29 [27.42, 31.16] SE 0.95^b^	34.61[32.71, 36.51] SE 0.96^c^	9.54 [7.68, 11.41] SE. 0.95^a^	<0.001	0.559	0.002
	ET (95)	8.92 [7.14, 10.71] SE 0.91^a^	26.13 [23.32, 28.94] SE 1.43^b^	33.36 [30.51, 36.21] SE 1.45^c^	14.96 [12.16, 17.77] SE 1.43^a^			
*p*-value		**0.012**	0.093	0.511	**0.004**			
Protein (%TE)	MT-IT (189)	2.40 [2.15, 2.66] SE 0.13^a^	5.88 [5.52, 6.23] SE 0.18^b^	6.97 [6.60, 7.33] SE 0.19^c^	1.84 [1.51, 2.17] SE 0.17^a^	<0.001	0.718	0.007
	ET (95)	1.91 [1.53, 2.29] SE 0.19^a^	5.31 [4.78, 5.85] SE 0.27^b^	6.83 [6.28, 7.38] SE 0.28^c^	2.85 [2.36, 3.34] SE 0.25^a^			
*p*-value		0.052	0.117	0.706	**0.002**			
Carbohydrate (g)	MT-IT (189)	31.78 [28.45, 35.07] SE 1.68^a^	72.08 [67.14, 77.02] SE 2.51^b^	68.44 [63.50, 73.38] SE 2.51^c^	25.35 [20.96, 29.74] SE 2.23^a^	<0.001	0.185	<0.001
	ET (95)	23.95 [18.96, 28.93] SE 2.53^a^	70.15 [62.72, 77.57] SE 3.77^b^	75.87 [68.44, 83.30] SE 3.77^b^	41.26 [34.66, 47.85] SE 3.35^c^			
*p*-value		**0.019**	0.697	0.135	**<0.001**			
Carbohydrate (%TE)	MT—IT (189)	6.46 [5.80, 7.11] SE 0.33^a^	14.42 [13.57, 15.28] SE 0.44^b^	13.38 [12.61, 14.14] SE 0.39^b^	4.80 [4.06, 5.54] SE 0.38^a^	<0.001	0.021	<0.001
	ET (95)	4.96 [3.98, 5.94] SE 0.50^a^	14.02 [12.73, 15.31] SE 0.66^b^	14.98 [13.83, 16.13] SE 0.58^b^	7.89 [6.78, 9.00 SE 0.57^a^			
*p*-value		**0.023**	0.641	**0.037**	**<0.001**			
Fat (g)	MT—IT (189)	12.48 [10.94, 14.01] SE 0.78^a^	32.45 [30.28, 34.61] SE, 1.10^b^	35.86 [33.55, 38.16] SE 1.17^b^	11.61 [9.48, 13.74] SE 1.08^a^	<0.001	0.582	<0.001
	ET (95)	9.39 [7.08, 11.70] SE 1.17^a^	28.33 [25.08, 31.59] SE 1.65^b^	34.11[30.65, 37.58] SE 1.76^b^	17.98 [14.78, 21.19] SE 1.63^c^			
*p*-value		**0.046**	0.059	0.451	**0.003**			
Fat (%TE)	MT—IT (189)	5.50 [4.84, 6.16] SE 0.34^a^	14.15 [13.37, 14.93] SE 0.40^b^	15.67 [14.87, 16.47] SE 0.41^b^	4.97 [4.17, 5.76] SE 0.40^a^	<0.001	0.824	0.003
	ET (95)	4.39 [3.40, 5.39] SE 0.50^a^	12.87 [11.70, 14.05] SE 0.60^b^	15.26 [14.06, 16.46] SE 0.61^b^	7.50 [6.32, 8.69] SE 0.60^a^			
*p*-value		0.096	0.105	0.613	**0.002**			
Sugar (g)	MT—IT (189)	15.63 [13.86, 17.40] SE 0.90^a^	29.32 [26.85, 31.79] SE 1.26^b^	27.18 [24.67, 29.69] SE 1.28^b^	12.08 [9.89, 14.27] SE 1.11^a^	<0.001	0.675	<0.001
	ET (95)	9.70 [7.04, 12.36] SE 1.35^a^	28.70 [24.98, 32.42] SE 1.89^b^	29.77 [25.99, 33.54] SE 1.92^b^	18.26 [14.96, 21.55] SE 1.67^a^			
*p*-value		**<0.001**	0.803	0.305	**0.005**			
Fiber (g)	MT—IT (189)	4.51 [4.04, 4.99] SE 0.24^a^	7.95 [7.41, 8.48] SE 0.27^b^	8.05 [7.47, 8.63] SE 0.30^b^	2.25 [1.80, 2.70] SE 0.23^c^	<0.001	0.069	<0.001
	ET (95)	2.47 [1.75, 3.18] SE 0.36^a^	6.74 [5.93, 7.55] SE 0.41^b^	7.84 [6.97, 8.72] SE 0.44^b^	3.46 [2.78, 4.13] SE 0.34^a^			
*p*-value		**<0.001**	**0.026**	0.726	**0.008**			
MUFA (g)	MT—IT (189)	4.68 [4.15, 5.20] SE 0.27^a^	12.13 [11.26, 13.00] SE 0.44^b^	13.66 [12.73, 14.59] SE 0.47^b^	4.24 [3.39, 5.09] SE 0.43^a^	<0.001	0.360	<0.001
	ET (95)	2.70 [1.90, 3.49] SE 0.40^a^	10.53 [9.23, 11.84] SE 0.66^b^	13.03 [11.63, 14.42] SE 0.71^b^	6.78 [5.50, 8.06] SE 0.65^c^			
*p*-value		**<0.001**	0.068	0.495	**0.003**			
PUFA (g)	MT—IT (189)	2.12 [1.81, 2.44] SE 0.16^a^	4.71 [4.29, 5.12] SE 0.21^b^	4.80 [4.43, 5.17] SE 0.19^b^	1.44 [1.14, 1.73] SE 0.15^a^	<0.001	0.221	0.002
	ET (95)	1.34 [0.86, 1.81] SE 0.24^a^	3.84 [3.22, 4.47] SE 0.32^b^	4.69 [4.13, 5.25] SE 0.28^b^	2.15 [1.71, 2.60] SE 0.23^a^			
*p*-value		**0.013**	**0.040**	0.762	**0.016**			
SFA (g)	MT—IT (189)	5.21 [4.56, 5.86] SE 0.33^a^	12.29 [11.34, 13.24] SE 0.48^b^	13.62 [12.62, 14.62] SE 0.51^b^	4.67 [3.72, 5.63] SE 0.49^a^	<0.001	0.576	<0.001
	ET (95)	3.17 [2.20, 4.15] SE 0.50^a^	10.98 [9.55, 12.41] SE 0.73^b^	12.86 [11.35, 14.37] SE 0.77^b^	7.62 [6.18, 9.05] SE 0.73^c^			
*p*-value		**0.002**	0.173	0.449	**0.002**			
Cholesterol (mg)	MT—IT (189)	65.12 [52.34, 78.06] SE 6.53^a^	116.55 [102.89, 130.20] SE 6.94^b^	122.35 [112.57, 132.14] SE 4.97^b^	29.58 [23.12, 36.05] SE 3.29^c^	0.005	0.050	0.010
	ET (95)	35.34 [16.01, 54.68] SE 9.82^a^	102.50 [81.97, 123.03] SE 10.43^b^	99.52 [84.81, 114.23] SE 7.4^b^	48.60 [38.87, 58.32] SE 4.94^a^			
*p*-value		**0.021**	0.305	**0.021**	**0.004**			
Caffeine (mg)	MT—IT (189)	61.93 [51.05, 72.80] SE 5.52^a^	47.30 [39.28, 55.33] SE 4.08^a^	18.79 [14.23, 23.36] SE 2.32^b^	9.09 [6.32, 11.85] SE 1.40^c^	<0.001	0.077	0.001
	ET (95)	30.93 [14.59, 47.28] SE 8.31	37.42 [25.36, 49.48] SE 6.13^ab^	22.85 [15.99, 29.70] SE 3.48^ab^	12.75 [8.60, 16.91] SE 2.11^c^			
*p*-value		**0.005**	0.220	0.376	0.187			
Alcohol (g)	MT—IT (189)	0.001 [−9.99, 0.002] SE 0.00^a^	0.32 [−0.03, 0.66] SE 0.18^a^	3.72 [2.66, 4.79] SE 0.54^b^	1.52 [0.65, 2.40] SE 0.44^c^	0.012	0.933	0.315
	ET (95)	0.00 [−0.001, 0.001] SE 0.001^a^	0.36 [−0.17, 0.88] SE 0.26^a^	2.79 [1.19, 4.39] SE 0.81^b^	2.26 [0.95, 3.58] SE 0.67^b^			
*p*-value		0.556	0.911	0.384	0.398			

Repeated measures two-way ANCOVA was performed, with the within-subject factor as “time windows” and the between-subject factor as “chronotype.” Covariates appearing in the model are evaluated at the following values: Ethnicity = 1.45, Age = 28.54, Deprivation Index = 5.73. Error bars: ±2 standard error (SE). Main effects were corrected using Greenhouse–Geisser estimates. Bonferroni-type adjustments were made on multiple comparison tests. Means in a row with different superscripts (^abcd^) differ significantly. All *p*-values for between-chronotype differences (within each window) are considered significant at a level of *p* < 0.05 and are shown in bold. Missing values: MT-IT *n* = 1; ET *n* = 2. SFA, saturated fatty acids; PUFA, poly-unsaturated fatty acids; MUFA, mono-unsaturated fatty acids; MT-IT, morning and intermediate types combined group; MT, morning types; ET, evening types; TE, total energy.

### 3.4 Chronotype and dietary intake across the four time windows within body composition groups

We next assessed in more detail whether chronotype was associated with differences in dietary intake between the high and low BF% and AG-ratio groups (after controlling for age, deprivation index, and ethnicity). This analysis was performed separately for each time window. Significant differences were observed between chronotypes for energy and nutrients, using ET as the reference category (Table 4; see also Figures 2a–d). For the late night/early morning window, we found that in the high BF% group, ET had lower energy, protein, fat, and carbohydrate intakes than MT-IT (*p* < 0.05; Table 4), but this was not observed in the normal BF% group. When adjusting for energy, fat (%TE) was no longer significantly different (data not shown). In the late morning/early afternoon window, ET with normal BF% had significantly lower energy, protein, and fat intakes than MT-IT. In contrast, in the high BF% group in the late evening/early night intake window, the ET had significantly higher energy, carbohydrate, and fat intake than MT-IT. Among participants with a normal BF% in the same window, ET also had a higher energy, protein, and carbohydrate intake (*p* < 0.05; Table 4) than MT-IT. When additionally controlling for energy (results not shown), there was no longer a significant association between fats and chronotype in the late night/early morning window. However, in the late evening/early night window, ET was also significantly associated with higher fat (%TE) than MT-IT in the normal BF% group.

**Table 4:** Associations between chronotype (ET vs. MT-IT) and dietary intake according to four intake windows using multiple linear regression, stratified by BF% and AG ratio groups.

Chronotype (ET as reference category-independent variable as predictor) for each of the following nutrient variables (as dependent variables), within the different anthropometric groups:												
Dependent variables	Normal BF% (*n* = 137)			High BF% (*n* = 150)			Normal AG ratio (*n* = 95)			High AG ratio (*n* = 192)		
	β	95% CI	*R^2^*	β	95% CI	*R^2^*	β	95% CI	R^2^	β	95% CI	*R^2^*
Late night/early morning intake (03:00–9:59 h)												
Energy (kJ)	−188	−554; 178	0.13 ^¥^	**−413**	**−720; −106**	0.11*	−188	−665; 289	0.08	**−327**	**−600, −54.5**	0.11¥*
Protein (g)	−1.38	−5.14; 2.38	0.09^¥^	**−4.45**	**−7.39; −1.51**	0.15*	−2.07	−7.07; 2.93	0.03^‡^	**−3.14**	**−5.77, −0.51**	0.13¥*
Fat (g)	−2.22	−7.06; 2.61	0.09 ^¥^	**−3.91**	**−7.79; −0.02**	0.11¥*	−2.99	−9.16; 3.18	0.07	−2.88	−6.38; 0.62	0.09 ^¥^
Carbohydrate (g)	−4.22	−13.9; 5.47	0.10 ^¥^	**−10.7**	**−19.6; −1.85**	0.06*	−1.44	−14.9; 12.0	0.04^‡^	**−9.38**	**−16.9, −1.83**	0.06*
Late morning/early afternoon (10:00–14:59 h) intake												
Energy (kJ)	**−587**	**−1,054; −119**	0.05¥*	−34.6	−505; 436	−0.24^‡^	**−786**	**−1,371; −200**	0.08^¥*^	−75.5	−479; 328	−0.02^‡^
Protein (g)	**−7.32**	**−12.9; −1.70**	0.04¥*	−0.50	−5.42; 4.43	−0.01^‡^	**−10.6**	**−17.4; −3.71**	**0.09** ^¥*^	−0.82	−5.23; 3.58	−0.01^‡^
Fat (g)	**−9.57**	**−15.9; −3.24**	0.05¥*	−0.61	−6.39; 5.17	<0.01^‡^	−10.5	−18.7; −2.25	0.05^¥‡*^	−2.06	−7.08; 2.97	<0.01^‡^
Carbohydrate (g)	−5.09	−19.3; 9.10	0.06^¥^	−0.03	−13.7; 13.7	<0.01^‡^	−11.0	−29.6; 7.54	0.06^¥‡^	1.09	−10.6; 12.8	0.02^¥‡^
Late afternoon/early evening (15:00–19:59 h) intake												
Energy (kJ)	29.6	−458; 517	0.27 ^Ᵹ‡^	13.0	−501; 527	−0.02^‡^	226	−433; 885	0.03^Ᵹ‡^	−57.9	−483; 367	−0.01^‡^
Protein (g)	−3.30	−8.62; 2.01	0.04^‡^	0.40	−4.89; 5.70	<−0.01^‡^	−1.57	−8.00; 4.86	0.02^‡^	−1.17	−5.80; 3.46	0.01^‡^
Fat (g)	−1.63	−6.05; 6.38	<0.01^¥‡^	−3.02	−9.72; 3.66	<0.01^‡^	3.43	−5.09; 12.0	<−0.01^‡^	−3.49	−8.97; 2.00	0.01^‡^
Carbohydrate (g)	7.08	−6.61; 20.8	0.0.1^Ᵹ^	7.88	−6.15; 21.90	<0.01^‡^	11.5	−8.56; 31.5	0.12^Ᵹ^	5.96	−5.31; 17.2	0.01^‡^
Late evening/early night intake (20:00–02:59 h)												
Energy (kJ)	**650**	**110; 1190**	0.18¥*	**603**	**111; 1094**	0.17^¥*^	418	−169; 1,004	0.20^¥^	**684**	**233, 1,135**	0.16*
Protein (g)	**6.12**	**0.92; 11.3**	0.13*	5.21	−0.06; 10.5	0.17^¥^	3.43	−2.48; 9.34	0.11	**6.01**	**1.38, 10.6**	0.16^¥*^
Fat (g)	5.03	−0.87; 10.9	0.15 ^¥^	7.25	**1.19; 13.3**	0.13^¥*^	2.69	−3.97; 9.35	0.17^¥^	**7.50**	**2.18, 12.8**	0.13*
Carbohydrate (g)	**19.9**	**6.94; 32.8**	0.24¥*	**12.9**	**0.94; 24.9**	0.18^¥*^	**14.2**	**0.92; 27.5**	0.27*	**16.3** ^**¥**^	**5.22, 27.4**	0.18*

^#^All models in this row had overall model significance (*p* < 0.05); significant p’s are shown in bold. *p < 0.05; Multiple linear regression models repeated within normal (<35%) and high (≥35%) BF% groups and then within normal AG Ratio (<0.8) and high AG ratio (≥0.8) groups. Dependent variable: Nutrient intake, Independent variable: Chronotype (ET as reference category coded as 1 and MT-IT as non-reference category coded as 0), adjusted for age, deprivation index and ethnicity; ^¥^Covariate Ethnicity significant in the model; Missing deprivation index (*n* = 3). ^‡^Covariate Deprivation score significant in the model. ^Ᵹ^Covariate Age significant in the model. BMI, body mass index; ß, beta; SE ( β ), standard error beta; CI, confidence interval; *R*^2^, adjusted *R*^2^; BF%, body fat percentage; AG, android-to-gynoid fat percentage ratio.

In the next step, dietary intake data were also stratified by the AG ratio to examine fat distribution and central adiposity more closely, while controlling for age, deprivation, and ethnicity within each time window (Table 4). For the late night/early morning intake window within the high AG ratio group, ET had significantly lower energy, protein, and carbohydrate intake than MT-IT. In the late morning/early afternoon ET, a normal AG ratio had significantly lower intakes of energy and protein than MT-IT. However, in the high AG ratio group during the late evening/early night intake window, ET had significantly higher energy, protein, carbohydrate, and fat intake than MT-IT. When controlling for energy (data not shown), ET was also associated with a significantly higher protein intake (%TE) than MT-IT in those with a high AG-ratio. For the same analysis with high and low-BMI groups, see Supplementary Table S2.

### 3.5 Correlations between chronotype, biomarkers, and early morning and late evening energy intake

To assess whether chronotype is correlated with biomarkers, we performed correlation analyses between continuous midsleep times (MSFsc) and biomarkers for the late night/early morning and late evening/early night windows separately. Overall, earlier midsleep times (i.e., toward MT) were negatively correlated with higher total serum, HDL, and LDL cholesterol, HbA1c, and ghrelin concentrations (Table 5). Later midsleep (i.e., toward ET) was positively correlated with higher insulin, leptin, and triglyceride concentrations. Correlations within the two time windows showed that higher morning energy intake was positively correlated with higher HDL cholesterol and ghrelin, and lower HbA1c, insulin, and leptin concentrations. In contrast, higher evening energy intake was positively correlated with higher triglycerides, HbA1c, and insulin concentrations, and negatively correlated with lower ghrelin concentrations.

**Table 5:** Correlations of individual chronotype (midsleep times) with biomarkers and early morning and late evening energy intake.

Variable	Midsleep time (MSFsc)	Late night/early morning energy intake	Late evening/early night energy intake
Early morning energy intake	−0.36***	—	−0.22***
Late evening energy intake	0.49***	−0.22***	—
Biomarkers			
Cholesterol (mmol/L)	−0.22***	0.09	−0.07
LDL cholesterol (mmol/L)	−0.15**	0.01	−0.05
HDL cholesterol (mmol/L)	−0.23***	0.23***	−0.07
Triglycerides (mmol/L)	0.19**	−0.07	0.14*
HbA1c (mmol/L)	−0.23***	−0.16**	0.26***
Insulin liggins (uU/ml)	0.26***	−0.19***	0.21***
Glucose (mmol/L)	0.08	−0.06	0.10
Ghrelin (pg/mL)	−0.29***	0.20***	−0.21***
Leptin (pg/mL)	0.16**	−0.18**	0.05
Peptide YY (pg/mL)	0.01	−0.03	0.45
C-reactive protein (mg/L)	−0.03	−0.04	−0.09

Pearson’s correlation analysis, *p < 0.05, **p < 0.01, ***p ≤ 0.001. HbA1c, hemoglobin A1C; LDL, low-density lipoprotein; HDL, high-density lipoprotein; FM, fat mass, FFM, fat-free mass; MSFsc, midsleep time corrected for midsleep on free days, assessed by the Munich Chronotype Questionnaire (MCTQ); continuous MSFsc values were used from all chronotype groups.

Additional correlation analyses revealed that those with later chronotypes (toward ET) were positively correlated with a higher body composition, particularly weight, BMI, BF mass, and hip circumference whereas those with earlier chronotypes (toward MT) that were inversely correlated with lower body composition parameters (all) except waist-to-hip-ratio and waist-to-height-ratio (see Supplementary Table S3).

## 4 Discussion

This study found that late chronotypes (ET) had higher BMI, BF%, and AG Ratio than earlier chronotypes (MT-IT), as well as poorer glucose homeostasis (higher HbA_1c_ and insulin), endocrine regulator markers (higher leptin and lower ghrelin), and some lipid profile outcomes (lower HDL). We also showed that ET was associated with lower energy, carbohydrate and protein intake in the late night/early morning window, but only for the high BF% group, which was reversed in the late evening/early night (with also higher fat intake), for example, after 20:00 h. Compared with the earlier chronotypes, the ET had the lowest micronutrient intakes, as evidenced by low intakes of folate, vitamins A, C, and E, magnesium, potassium, calcium, riboflavin, and iodine, which is comparable to other study reports (7, 15). The low micronutrient intakes seen in the ET in other studies were suggestive of low intake of micronutrient-dense and antioxidant-rich foods such as fruit, vegetables, wholegrains, and dairy (8, 11, 13, 17, 21, 61–63). As the ET in this study mostly comprised women of Pacific ethnicity, these findings are consistent with previous reports of higher consumption of energy-dense foods and not likely meeting vegetable and fruit intake guidelines (64) in Pacific people. For example, another NZ study among healthy young NZ women (65) showed that Pacific women were more likely to have a “refined and processed” dietary pattern, alongside a profile of higher BMI, excess adiposity, high insulin and leptin concentrations, which may exacerbate the deposition of glucose and fatty acids into adipose tissue. In contrast, the diet in NZE women was characterized by a ‘sweet and savory snacking pattern, likely driven by energy and starchy foods. Both groups were less likely to follow the “fruit and vegetable” dietary pattern, which is consistent with the low micronutrient intakes seen in ET in this study. Jayasinghe et al. (65) also reported that Pacific women had higher plasma HbA1c and glucose concentrations than NZE women, a finding similar to our study. Furthermore, our study findings compare well with others, suggesting that the role of diet in the development of obesity is too simplistically described as energy intake not matching energy expenditure (66). The timing of food intake during the 24-h day and the associated food choices have been associated with weight gain and adverse metabolic health outcomes (36, 67–69).

### 4.1 Body composition

In this study, we also investigated a range of other body composition markers, which is important as BMI is not a good proxy of FFM and other body composition markers, particularly when making comparisons involving people of different ethnic origins (as is done in this study) with different body builds and body fat distributions. Previous research demonstrated that body composition profiles differed between Pacific and NZE women, with Pacific women having a 2.9 kg higher FFM than NZE women, whilst still having a greater absolute fat mass (70). The android-to-gynoid (AG) fat percentage ratio, assessed here for the first time among chronotypes, is rarely studied but is a more sensitive marker of cardiometabolic risk than BMI (71, 72). We found that women with a later chronotype (ET) were associated with higher BMI, BF%, and AG ratio than those with earlier chronotypes (MT-IT), despite the two chronotype groups having similar overall energy and macronutrient intakes. These results corroborate findings from a recent systematic review reporting that ET is often associated with an overweight or obese BMI profile with more unhealthy metabolic biomarkers, despite having equivalent energy and/or nutrient intakes to MT (73).

### 4.2 Macronutrient distribution

Chronotype is associated with differences in both sleep and wake patterns (6) and meal timing. The importance of timing dietary intake is supported by strong evidence showing that both macronutrient metabolism and energy expenditure differ across the 24-h light–dark cycle (29–35, 47–49), thereby affecting energy utilization and body composition over time (36–38). Previous research has shown that ET’s delay their evening meal to later clock times (main meals) (10, 19, 21, 23, 45, 62, 74). Studies examining the variances between chronotypes and the distribution of food intake throughout the day primarily reported on energy distribution (16–21) and, to a lesser extent, how macronutrients are distributed through daily intakes (17, 20, 22, 23). In our study, the ET had significantly higher carbohydrate intakes than MT, IT, or MT-IT, but no significant differences were found for protein or fat. In contrast, Günal et al. reported that females with IT consumed significantly more carbohydrates (g) than MT and ET. Both IT and MT consumed significantly more protein than the ET (75). There is growing evidence that later chronotypes consume more energy and macronutrients in the evening and less energy in the early morning compared with early chronotypes (16–19, 21–23). A non-chronotype US study that reported on the timing of food intake found that adults who had excessive energy intake at dinner compared with breakfast were associated with a higher obesity risk related to low-quality food choices (mainly carbohydrates and fat) (76). In our study, we saw comparable patterns between MT-IT and ET, with a significant interaction between chronotype and time of day on energy and macronutrient intake. The ET consumed only 9.01% of their total daily energy intake in the morning by 10:00 h vs. 15.3% for MT-IT. At night after 20:00 h, ET consumed 2.5 times that quantity (22.6%) vs. 1.5 times (11%) by MT-IT. This is in accordance with a Finnish study that found similar results with ET 14%, consuming less than MT 19%, of their total energy intake in the morning, and vice versa at night, ET 20% and MT 14% (17).

### 4.3 Chronotype-specific dietary distribution and body composition outcomes

Considering the macronutrient distribution of their diet, the MT-IT in this study had higher intakes of energy, protein, carbohydrate, and fat in the late night/early morning window compared with the ETs who had higher intakes of all these nutrients in the late evening/early night. When looking at these nutrient differences as %TE, fat and protein intakes were no longer significantly different between chronotypes in the late night/early morning window. Hence, adjusting nutrient intake for energy intake helps control for confounding arising from the association between energy intake and chronotype. However, fat and protein remained significantly higher in ET during the late evening/early night window, even when expressed as %TE, suggesting that these nutrient intakes were, in fact, proportionally higher in ET (rather than due to higher energy intake). These findings are consistent with previous research, suggesting that, beyond an internal preference for late sleep and wake timing (54), ET are also shifting their timing of food intake later in the day, into the late morning and into the evening (73).

Daytime is the most advantageous phase for acquiring and storing energy. This is evidenced by optimal circadian insulin sensitivity and glucose tolerance, along with increased energy expenditure, lipogenesis, and adiponectin synthesis (49). Conversely, during the fasting phase (which physiologically occurs at night while sleeping), there is a marked increase in gluconeogenesis, glycogenolysis, and lipolysis (49), resulting in elevated concentrations of circulating fatty acids, triglycerides, and cholesterol (48, 77). Since the ET eat closer to their bedtime, their fasting period is shortened, which may be associated with weight gain. When food is consumed later during the 24-h day, it may have a “desynchronizing” effect on circadian metabolic functions, which may potentially be associated with elevated adiposity levels and poor metabolic health outcomes (78–82). A possible explanation for the higher ET-dietary intake at night is the breakfast-skipping theory, which suggests that breakfast skipping is associated with greater hunger later in the day, particularly in the evening, which may result in overeating at the evening meal or excessive late-night snacking (83).

Few studies have examined the impact of chronotype-specific dietary distribution on body composition outcomes (20, 23). Xiao et al. (23) observed a higher BMI among ET with a lower morning and higher evening dietary intake pattern. They found that when MT consumed a higher proportion of their energy from carbohydrates and protein 2 h of waking up in the morning, they were less likely to be overweight/obese. When ET consumed a higher proportion of energy, carbohydrates, and protein in the evening, within 2 h before bedtime, they were more likely to be overweight/obese than the MT-IT (73), which is in line with the associations observed in the present study. However, their results showed stronger associations for protein and carbohydrate intake than for fat intake, whereas in our study, ET had higher energy, fat, and carbohydrate intake in the high BF% group. When looking at the nutrients expressed as %TE, associations with fat were no longer observed in the late night/early morning window in the high BF% group, but associations with protein and carbohydrates remained. In the late evening/early night window, all associations between chronotype and nutrients remained after controlling for energy (%TE), suggesting that, apart from fat in the late night/early morning window between carbohydrates and protein were independent of energy intake. To clarify these findings, the distribution of body fat compartments was assessed using the AG ratio, a more sensitive predictor of non-communicable diseases and metabolic syndrome in healthy adults than BMI or fat mass (71, 72, 83). Again, when comparing ET with MT-IT in the high AG ratio group (more central body fat), ET demonstrated lower energy, protein, and carbohydrate intakes in the late night/early morning, whereas it showed higher energy, protein, carbohydrate, and fat intakes in the late evening/early night. However, this was not seen within the normal AG-ratio group (apart from carbohydrates), suggesting that late-night eating among ET may be associated with greater central obesity.

All associations remained significant after controlling for %TE, and protein was also significantly higher in the normal AG-ratio group during the late afternoon/early night window. While the adjusted data may suggest that protein and carbohydrates are not responsible for a higher AG-ratio, other factors, such as the “quality” of nutrients, may be relevant. For example, complex vs. simple carbohydrates and plant vs. animal proteins may potentially be “canceling out” opposing effects (which have not been explored in this study). Dietary intakes differ significantly between ethnic groups in NZ, with Pacific people having lower intakes of micronutrient-dense foods such as fruit and vegetables, while having higher intakes of nutrient-poor foods such as takeaways and sugar-sweetened beverages (84). Furthermore, since the majority of the ET in this study were of Pacific ethnicity, their lower micronutrient intakes may also have been attributed to limited access to nutrient-dense foods; the ET in this study had a significantly higher deprivation index score compared to MT-IT.

### 4.4 Metabolic biomarkers

In this study, we found that ET had higher plasma concentrations of triglycerides, leptin, insulin, and HbA1C compared to the MT-IT. These findings are in line with the systematic review and meta-analysis by Lotti et al. (85), which reported that ET was associated with higher blood glucose, HbA1c, LDL cholesterol, and triglyceride concentrations and therefore demonstrated worse cardiometabolic risk outcomes. Consistent with our findings, the review did not report differences in total energy intake, insulin, HDL cholesterol, or total cholesterol between chronotype groups. This unfavorable cardiometabolic-ET profile may be related to ethnicity, as the ET comprised mainly Pacific women. There is some evidence that Pacific peoples are more susceptible to elevated plasma HbA1c concentrations and impaired glucose homeostasis, and to associated metabolic diseases when compared with NZE (86). In a separate analysis of PROMISE study data, we found that Pacific women and women with high BF% had significantly higher fasting plasma insulin and HbA1c concentrations than NZE women and women with low BF%, respectively (52). This was also confirmed in another NZ study investigating metabolic disease risk among different ethnic groups in relation to sedentary behavior (87). Both studies were conducted in healthy women, and thus the chronic pathological response was not evident; HbA1c concentrations were normal and below 41 mmL/L (33.0 ± 3.1 in Pacific women in the PROMISE study (52) and 30.8 ± 3.2 mmol/L in Pacific women in the EXPLORE study (87). Later timing of food intake has also been linked with poor metabolic health (as is android obesity). In this study, late eaters who consumed more energy in the evening were positively correlated with a later chronotype (ET), higher body composition (including weight, BMI, HC, and body fat mass), poorer glucose homeostasis (higher HbA1c and insulin), and higher triglyceride concentrations. In contrast, early eaters who consumed more energy in the morning were associated with an earlier chronotype (MT), lower body composition, and metabolic markers. Garaulet et al. (88) found that women with lower body fat experienced metabolic changes (including reduced glucose tolerance, lower resting energy expenditure, and lower carbohydrate oxidation after one week of consuming lunch at a late time, similar to that characteristically observed in women with higher body fat (88). These findings suggest that metabolic alterations may occur when food is consumed at an inappropriate time of the day. Several physiological mechanisms may explain the less favorable metabolic profile as well as the higher body composition profile observed in the ET group. Circadian disruption in muscle and hepatic tissues occurs in opposite directions during the day (improved in the morning prior to the period of feeding and activity and reduced in the evening prior to the period of fasting and inactivity) (57). This may impair insulin signaling, glucose metabolism, and insulin sensitivity as well as alter lipid handling. Higher carbohydrate tolerance and insulin sensitivity in the morning compared to the evening have been reported, even with similar carbohydrate loads, as well as less efficient fat oxidation at nighttime. Outcomes include insulin resistance and central adiposity, which are typical of ET (57, 89, 90).

### 4.5 Strengths and limitations

Strengths of this study are the in-depth exploration of several body composition parameters and metabolic biomarkers between chronotypes in healthy women. Previous research was mostly in participants with overweight or obesity, often with underlying health conditions. We also conducted a comprehensive, in-depth assessment of total energy, macronutrient, and micronutrient intake, with a particular focus on the distribution of dietary intake throughout the day and its subsequent impacts. The comprehensive assessment of dietary intake using 5-day food records collected on non-consecutive weekdays and weekend days, provided an excellent variety of food intake within and between participants. Food records were meticulously reviewed and discussed with each participant by dietitians to ensure the accuracy of the dietary data. As this was a self-reported measure at home, a limitation is that misreporting and recall bias during the interviews are always possible. This may be more so among ETs who are known to exhibit irregular eating patterns and behaviors, potentially increasing errors in dietary data. Another limitation of this study is the lack of an internal circadian phase marker in our study population; there is no indication of alignment (or misalignment) between any of the tested variables with the internal circadian phase of the central biological clock (e.g., assessed by the DLMO) and with peripheral biological clocks. Subsequently, meal timing was interpreted relative to external clock time rather than individual biological timing. Hence, we can only speculate that eating at later clock times is detrimental to body composition due to potentially misaligned glucose and lipid metabolism, or that eating, compared with skipping breakfast, can improve insulin sensitivity (57, 89). Qian and Vujovic (50) showed in simulated night shift work that when scheduled eating occurred during the daytime despite “working” on simulated night shifts, it was more beneficial for glucose tolerance and insulin sensitivity than scheduled eating at nighttime. Studies with large adult sample sizes revealed that higher BMI, body weight, or obesity was associated with late-night energy intake (after 21:00 h; *n* = 21,000) (91), and higher abdominal obesity with late meal intake (after 22:00 h; *n* = 7,379) (92).

This study included many repeated metabolic and food-related outcomes. Although the observed effect sizes were relatively small, the clinical relevance of these findings, along with their potential health implications and associated healthcare costs, is substantial. This is supported by a growing body of literature in the field, as discussed (93). Because these findings were hypothesis-driven, consistent across related yet distinct measures within the same time windows, and supported by the literature, they represent stronger, converging evidence rather than chance associations. For example, associations showed that higher intake by ET in the late night/early morning was inversely associated with BF% and AG-ratio, while higher intake by ET in the late evening/early night was associated with higher BF% and AG-ratio. Metabolic markers HbA1C and insulin supported this finding, being associated with higher intake in the late evening/early night window, and the results are in line with findings of other studies (57, 92).

Our study population comprised only young, healthy women; however, blood samples (only one early morning sample) and other data were collected without consideration of menstrual cycles. Menstrual cycle hormones may have confounding effects on taste perception, hunger, energy intake (a progressive decrease in eating during the follicular phase; higher intakes during the luteal phase), and energy expenditure (94, 95). Given the known age-dependent statistical distribution of chronotypes (56), we expected that our cohort of healthy, young women would include only a small number of MT. This was not controlled for in the study’s original design, and a random cross-sectional recruitment strategy was used. Further, although this study was originally designed to include equal numbers of participants from two ethnicities with different body compositions, it was not specifically designed to have an equal number of MTs and ETs across those two ethnic groups. Furthermore, the small sample size of MT in both the Pacific and NZE groups prevented stratification by ethnicity. While combining MT and IT-groups strengthened statistical interpretations regarding ET compared with the rest of the sample due to small numbers in MT, the specific metabolic implications of IT could not be explored in more depth independently of MT. However, our approach is supported by previous evidence of more favorable metabolic and body composition profiles associated with morningness as opposed to eveningness (57, 96).

Ethnicity is known to influence dietary intake, body composition, and metabolic profiles through a variety of factors, including cultural, socioeconomic, religious, and behavioral factors (97). Therefore, observed differences between chronotype groups should be interpreted with caution as the chronotype categories substantially overlapped with ethnicity in this study. Despite adjusting for ethnicity and deprivation in our analyses, the high proportion of Pacific participants in the ET group means that our findings may be biased, in part, by interspersed ethnicity-related factors such as dietary habits and lifestyle differences. As a result, the ability to fully isolate the effect of chronotype from ethnicity is limited, and caution is needed when generalizing these results to more ethnically diverse populations. There is only sparse evidence from the literature on chronotypes across different ethnicities in NZ, and the results are mixed. Some data come from large New Zealand surveys comparing NZ European, Māori, Pacific, and Asian adolescents. The study by Galland et al. showed that Pacific adolescents had bedtimes 41 min later than those of NZ Europeans (*n* = 4,192) (98). The authors interpreted this as socially, behaviorally, and environmentally driven rather than biological claims between ethnicities. There is some information on specific sleep habits among Pacific ethnicities, which includes collectivist family structure, community obligations, crowded housing, shift work, and socioeconomic conditions, which were described as determinants of Pacific sleep timing, which may differ from European NZ sleep habits (99). In contrast, Paine et al. found that morningness/eveningness preference was largely independent of ethnicity, gender, and socioeconomic position in NZ. They proposed that, chronotype is rather attributable to endogenous factors (100).

In addition, whether chronotype leads to different health outcomes between the two ethnic groups is not well known. Hence, future research should conceptualize chronotype as a more multidimensional trait shaped not only by circadian phase and sleep patterns, but also by light exposure, cultural norms, work obligations, socioeconomic conditions, and potentially population genetics, as suggested in a recent review (101). Such an approach would help disentangle whether the observed differences are driven by chronotype itself or by underlying ethnic and cultural influences, personality traits, or social and work-related constraints affecting sleep and dietary habits.

## 5 Conclusion

In conclusion, all chronotype groups consumed on average similar amounts of total energy and macronutrients. However, in ET, food intake was lower in the late night/early morning window and higher in the late evening/early night window, which was associated with greater obesity profiles, less favorable metabolic biomarker profiles, and lower micronutrient intake in ET women. This eating pattern may reflect food intake, digestion, and metabolism during the normal fasting phase of the light/dark cycle, leading to storage rather than utilization. The MT-IT women’s habitual early wake-, sleep and meal-timing may therefore be associated with healthier body composition and metabolic biomarker profiles.

From this, it follows that a variety of factors are crucial for health outcomes. It appears that not only what and when we eat, but also when we sleep relative to the external day-night cycle and how these lifestyle factors align with metabolic health outcomes are important. Therefore, there is a need to explore further the link between “chrono-nutrition” and obesity outcomes, particularly in ethnically diverse populations, to better distinguish the independent effects that chronotype may have and to tailor personalized health interventions in the future.

## Data Availability

The raw data supporting the conclusions of this article will be made available by the authors, without undue reservation.
