# Supplementary material for: Chronotype and associations with dietary intake, meal timing, body composition, and metabolic biomarkers
Source: Front Nutr. 2026 Jul 7;13:1862060. doi: 10.3389/fnut.2026.1862060 (PMC13387395; doi:10.3389/fnut.2026.1862060)
Supplement: Supplementary file 1 [file Supplementary_File_1.docx]

Supplementary Material

# Supplementary Figures and Tables

**Supplementary Figure 1:** Average hourly energy intakes.

**
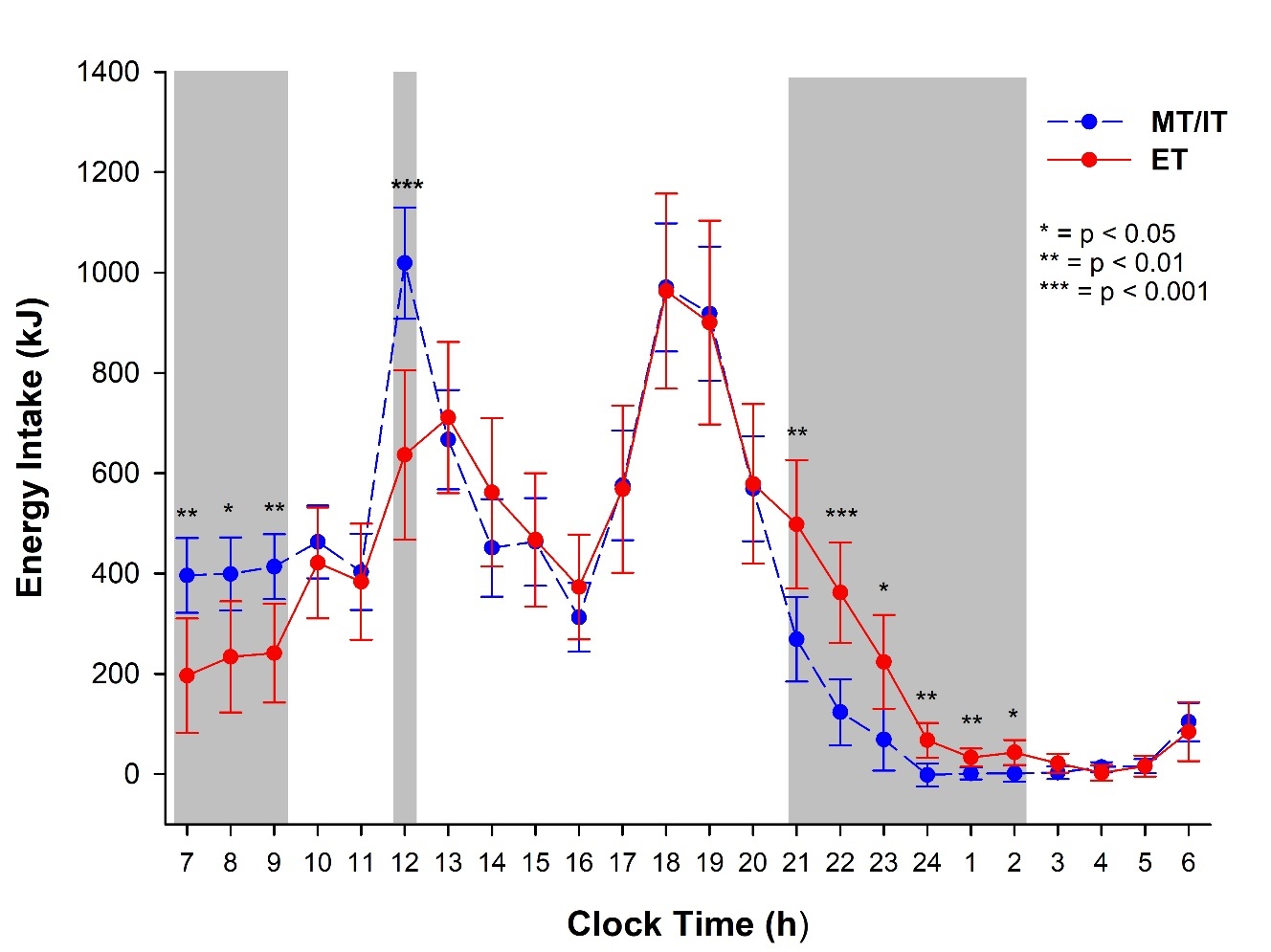
**

**Figure Legend:** Averaged hourly intakes for morning and intermediate chronotypes (MT-IT; blue symbols and dashed lines) and for evening types (ET; red symbols and lines). Estimated marginal means are shown ± 2 Standard Errors (SE); * *p* <0.05; ** *p* < 0.01; *** *p* < 0.001; the grey rectangles highlight the time bins with signficant differences between both chronotype groups. Morning type (MT; n=35) and Intermediate chronotype (IT; n=155) were combined as a group (MT-IT), Evening type (ET; n=97).
