# Supplementary material for: Chronotype and associations with dietary intake, meal timing, body composition, and metabolic biomarkers
Source: Front Nutr. 2026 Jul 7;13:1862060. doi: 10.3389/fnut.2026.1862060 (PMC13387395; doi:10.3389/fnut.2026.1862060)
Supplement: Supplementary file 2 [file Supplementary_File_2.docx]

Supplementary Material

# Supplementary Figures and Tables

Supplementary Figure 2A-D: Distribution of Energy, protein, carbohydrate and fat intake relative to individual midsleep time (6-hour windows)


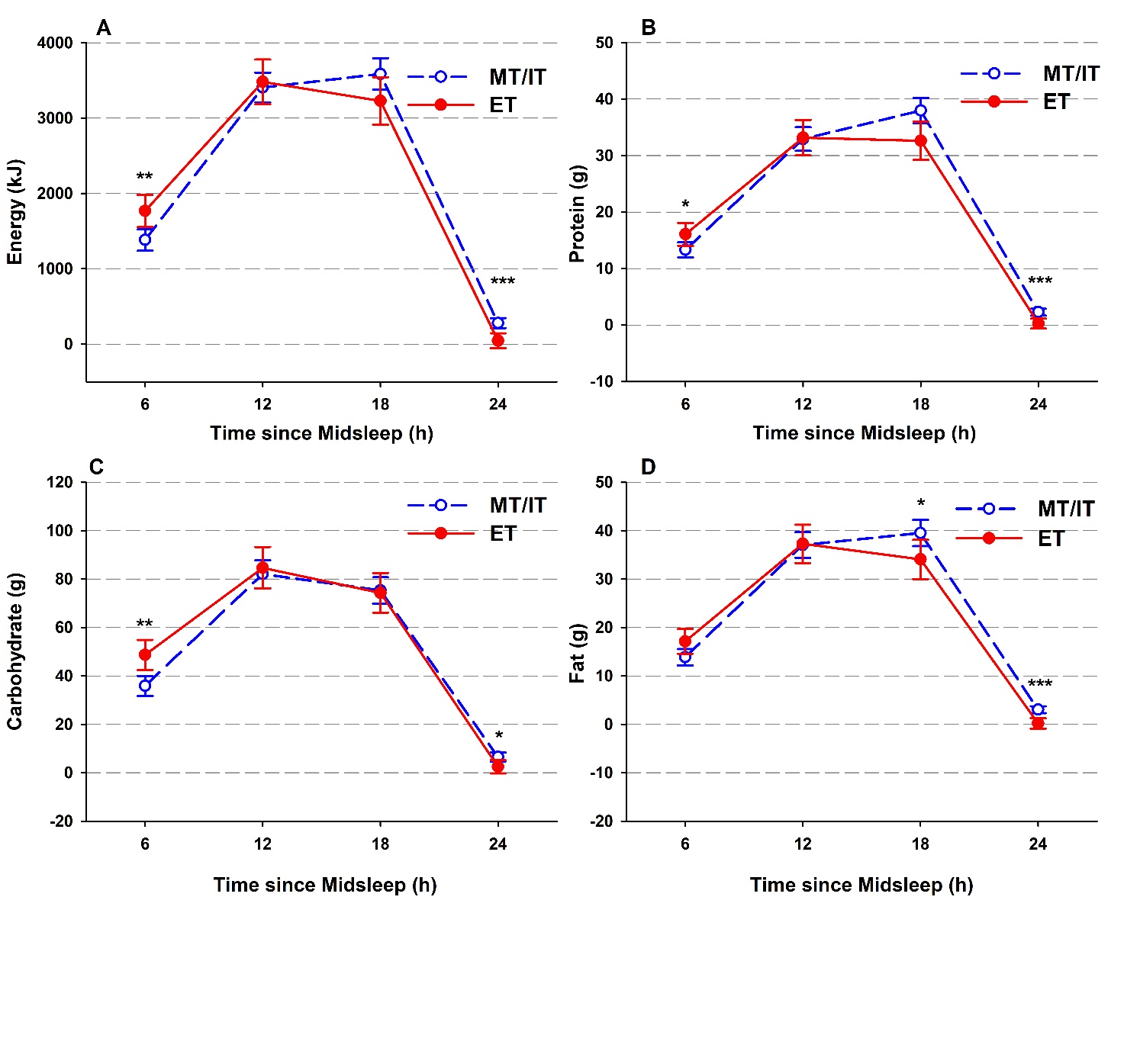


**Figure Legend:** 6-hour time bins relative to midsleep are shown for combined morning and intermediate types (MT/IT; dashed blue lines and open blue circles and for ET; solid red lines, red circles); the data shows estimated marginal means, ± 2 Standard Errors (SE). *P < 0.05, **P < 0.01, ***P < 0.001. Chronotypes morning type (MT) and intermediate type (IT) were combined as a group (MT-IT), Evening type chronotype (ET).
