# Supplementary material for: Chronotype and associations with dietary intake, meal timing, body composition, and metabolic biomarkers
Source: Front Nutr. 2026 Jul 7;13:1862060. doi: 10.3389/fnut.2026.1862060 (PMC13387395; doi:10.3389/fnut.2026.1862060)
Supplement: Supplementary file 3 [file Table_1.docx]

Supplementary Material

# Supplementary Figures and Tables

**Supplementary Table 1** Distribution of dietary intake according to habitual midsleep time (four 6-h time windows) for different chronotype groups

| **Dietary intake** | **CT**  **(n)** | **6 hrs past midsleep** | **12 hrs past midsleep** | **18 hrs past midsleep** | **24 hrs past midsleep** | **P-values; main effects:** | | | |
| --- | --- | --- | --- | --- | --- | --- | --- | --- | --- |
|  |  |  |  |  |  | **Time** | | **CT** | **Time*CT** |
| Energy (kJ) | MT-IT (190) | 1381 [1241, 1520]^a^  SE 71.00 | 3405 [3210, 3600]^bc^  SE 99.02 | 3584[3378, 3790]^bc^  SE 104.61 | 278.2 [213.6, 342.9]^d^  SE 32.85 | **<0.001** | 0.705 | | **0.007** |
|  | ET (97) | 1767 [1557,1997]^a^  SE 106.75 | 3480 [3187, 3773]^bc^  SE 148.88 | 3227[2917, 3536]^bc^  SE 157.28 | 43.82 [-53.39, 141.03]^d^  SE4 49.39 |  |  |  |  |
| P-value* |  | **0.006** | 0.70 | 0.08 | **<0.001** |  |  | |  |
| Protein (g) | MT-IT (190) | 13 32 [12.00, 14.64]^a^  SE 0.67 | 32.91 [30.85, 34.98]^b^  SE 1.05 | 37.95 [35.74, 40.17]^c^  SE 1.13 | 2.28 [1.66, 2.87]^d^  SE 0.30 | **<0.001** | 0.206 | | **0.010** |
|  | ET (97) | 16.05 [14.07, 18.04]^a^  SE 1.01 | 33.17 [30.07, 36.27]^bc^  SE 1.58 | 32.61 [29.28, 35.94]^bc^  SE 1.69 | 0.24 [0.64, 1.12]^d^  SE 0.45 |  |  |  |  |
| P-value* |  | **0.04** | 0.90 | 0.17 | **<0.001** |  |  | |  |
| Carbo-hydrate (g) | MT-IT (190) | 35.86 [31.79, 39.93]^a^  SE 2.07 | 82.02 [76.38, 87.67]^bc^  SE 2.87 | 75.29 [69.95, 80.63]^bc^  SE 2.72 | 6.55 [4.76, 8.34]^d^  SE 0.91 | **<0.001** | 0.309 | | **0.037** |
|  | ET (97) | 48.67 [42.55, 54.86]^a^  SE 3.11 | 84.66 [76.17, 93.14]^bc^  SE 4.31 | 74.29 [66.25, 82.32]^bc^  SE 4.08 | 2.49 [-0.21, 5.19]^d^  SE 1.37 |  |  |  |  |
| P-value* |  | **0.002** | 0.642 | 0.851 | **0.025** |  |  | |  |
| Fat (g) | MT-IT (190) | 13.86 [12.19, 15.54]^a^  SE 0.85 | 37.02 [34.43, 39.61]^bc^  SE 1.32 | 39.51 [36.86, 42,17]^bc^  SE 1.35 | 3.04 [2.34, 3.74]^d^  SE 0.36 | **<0.001** | 0.229 | | **0.019** |
|  | ET (97) | 17.13 [14.61, 19.65]^a^  SE 1.28 | 37.28 [33.83, 41.18]^bc^  SE 1.98 | 34.06 [30.06, 38.05]^bc^  SE 2.03 | 0.20 [-0.86, 1.25]^d^  SE 0.54 |  |  |  |  |
| P-value |  | 0.052 | 0.921 | **0.041** | **<0.001** |  |  | |  |

CT, chronotype; MT-IT, morning- and intermediate types combined group; MT, morning types; ET, evening types. Repeated measures Two-way ANCOVA performed using the within subject factor as "time windows" and between subject factor as "chronotype". Covariates appearing in the model are evaluated at the following values: Ethnicity = 1.45, Age = 28.54, Deprivation Index = 5.73 +/- 2 SE. Main effects were corrected using Greenhouse-Geisser estimates. Bonferroni type adjustments were made on multiple comparison tests. Estimated marginal means in a row with different superscripts (^abcd^) differ significantly. *p* -values for between-chronotype differences (within each window) are considered significant at a level of  *p* < 0.05, shown in **bold**. Missing values: MT-IT n = 1; ET n = 2.
