# Supplementary material for: Chronotype and associations with dietary intake, meal timing, body composition, and metabolic biomarkers
Source: Front Nutr. 2026 Jul 7;13:1862060. doi: 10.3389/fnut.2026.1862060 (PMC13387395; doi:10.3389/fnut.2026.1862060)
Supplement: Supplementary file 4 [file Table_2.docx]

Supplementary Material

# Supplementary Figures and Tables

**Supplementary Table 2.** The association between chronotype (ET vs MT-IT) and dietary intake according to four intake windows using multiple linear regression, stratified by BMI ratio groups

| Chronotype (ET as reference category - independent variable as predictor) for each of the following nutrient variables (as dependent variables), within the different anthropometric groups | | | | | | |
| --- | --- | --- | --- | --- | --- | --- |
| **Dependent variables** | **Normal BMI**  **(n = 111)** | | | **High BMI**  **(n = 176)** | | |
|  | $\beta$ | 95% CI | *R^2^* | $\beta$ | 95% CI | *R^2^* |
| Late Night/Early Morning Intake (before 10:00): | | | | | | |
| Energy (kJ) | -346 | -728; 36.3 ^¥^ | 0.23 | -285 | -585; 14.2 | 0.09 |
| Protein (g) | **-3.78** | **-7.47; -0.09 ^¥^** | **0.22** | -2.60 | -5.65; 0.45 | 0.09 |
| Fat (g) | -3.56 | -8.48, 1.36 ^¥^ | 0.19 | -2.78 | -6.67; 1.11 ^¥^ | 0.85 |
| Carbohydrate (g) | -7.74 | -18.7, 3.19 ^¥^ | 0.16 | -7.76 | -15.9, 0.40 | 0.06 |
| Late morning/early afternoon (10:00-14:59) intake: | | | | | | |
| Energy (kJ) | **-539** | **-978; -99.3^¥^** | **0.12** | -109 | -564; 345 | -0.005 |
| Protein (g) | **-5.91** | **-11.8; -0.7** | **0.08** | -1.8 | -6.52; 3.00 | -0.02 |
| Fat (g) | **-7.89** | **-13.9; -2.89^¥^** | **0.10** | -2.21 | -7.94; 3.53 | 0.03 |
| Carbohydrate (g) | -6.47 | -19.7; 6.72 | 0.09 ^¥^ | -0.16 | -13.4; 13.7 | 0.04 |
| Late afternoon/early evening (15:00-19:59) intake: | | | | | | |
| Energy (kJ) | -51.5 | -569; 466^‡^ | 0.08 | 31.9 | -442; 506 | <0.01 |
| Protein (g) | -3.82 | -9.56; 1.92 | 0.08 | -0.12 | -4.99;4.75 | 0.34 |
| Fat (g) | -0.87 | -7.65; 5.92^‡^ | 0.04 | -2.4 | -8.52; 3.68 | 0.03 |
| Carbohydrate (g) | 7.21 | -6.87; 21.3^‡^ | 0.15 | 7.24 | -5.92; 40.4 | 0.03^‡^ |
| Late Evening/Early Night Intake (after 20:00): | | | | | | |
| Energy (kJ) | 425 | -87.7, 938 ^¥^ | 0.20 | **722** | **235, 1209 ^¥^** | **0.19** |
| Protein (g) | 3.75 | -1.45, 8.94 ^¥^ | 0.16 | **6.21** | **1.19, 11.2^¥^** | **0.17** |
| Fat (g) | 2.90 | -2.81, 8.60^¥^ | 0.17 | **8.10** | **2.30, 13.9** | **0.15** |
| Carbohydrate (g) | **13.1** | **1.01, 25.1^¥^** | **0.25** | **17.2** | **5.28, 29.1^¥^** | **0.21** |

BMI, body mass index; SE ($\beta$), standard error beta; CI, confidence interval; R^2^, adjusted R^2^. ^#^All models in this row had overall model significance (*p* <0.05); significant *p’s are* shown in **bold**. * *p* <0.05; Multiple linear regression models repeated within normal (<35 %) and high (≥35 %) BF% groups and then within normal AG Ratio (<0.8) and high AG Ratio (≥0.8) groups. Dependent variable: Nutrient Intake, Independent variable: Chronotype (ET as reference), adjusted for age, deprivation index & ethnicity; ^¥^Covariate Ethnicity significant in the model; Missing deprivation index (n = 3). ^‡^ Covariate Deprivation score significant in the model. *^Ᵹ^* Covariate Age significant in the model.
