# Supplementary material for: Chronotype and associations with dietary intake, meal timing, body composition, and metabolic biomarkers
Source: Front Nutr. 2026 Jul 7;13:1862060. doi: 10.3389/fnut.2026.1862060 (PMC13387395; doi:10.3389/fnut.2026.1862060)
Supplement: Supplementary file 5 [file Table_3.docx]

Supplementary Material

# Supplementary Figures and Tables

**Supplementary Table 3** Correlations of chronotype (continuous data with habitual midsleep times) with body composition and early morning energy intake (before 10:00) & late evening energy intake (after 20:00)

| **Body composition & metabolic biomarkers** | **Midsleep time (MSFsc)** | **Early morning energy intake** | **Late evening energy intake** |
| --- | --- | --- | --- |
| **Body composition markers** |  |  |  |
| Weight (kg) | 0.24*** | -0.19** | 0.16** |
| BMI | 0.20*** | -0.17* | 0.13* |
| Body fat mass (BIA, kg) | 0.22*** | -0.19** | 0.14* |
| Fat mass (DXA, kg) | 0.21*** | -0.18** | 0.11 |
| BF% (BIA) | 0.17** | -0.18** | 0.10 |
| BF% (DXA) | 0.14* | -0.16** | 0.04 |
| Visceral Fat % | 0.21*** | -0.20*** | 0.08 |
| Android Fat % | 0.20*** | -0.19*** | 0.08 |
| Gynoid Fat % | 0.14* | -0.14* | 0.31 |
| Android to Gynoid fat Ratio | 0.20*** | -0.19*** | 0.10 |
| FM to FFM Ratio (DXA) | 0.13* | -0.15* | 0.03 |
| FM to FFM Ratio (BIA) | 0.16** | -0.13* | 0.10 |
| Waist circumference | 0.15* | -0.13* | 0.08 |
| Hip circumference | 0.20*** | -0.17** | 0.15* |
| Waist-to-hip-ratio | 0.002 | -0.03 | -0.06 |
| Waist-to-height-ratio | 0.11 | -0.11 | 0.06 |
| Muscle mass (BIA) | 0.22*** | -0.17** | 0.15* |
| Lean body mass (DXA, kg) | 0.13* | -0.15* | 0.03 |

BMI, body mass index; BF%, body gat percentage; FM, fat mass; FFM, fat free mass; BIA, biooelectrical impedance analysis; DXA, dual-energy X-ray absorptiometry; MSFsc, Midsleep time on free days corrected for sleep duration; MCTQ, Munich Chronotype Questionnaire. Please note: a lower midsleep time (derived from continuous MSFsc values of the MCTQ) implies an earlier chronotype and vice versa. Pearson’s Correlation Analysis, *P < 0.05, **P < 0.01, ***P ≤ 0.001.
